# Comparison of contemporary invasive and non-invasive *Streptococcus pneumoniae* isolates reveals new insights into circulating anti-microbial resistance determinants

**DOI:** 10.1128/aac.00785-23

**Published:** 2023-10-12

**Authors:** Charlie Higgs, Lamali Sadeesh Kumar, Kerrie Stevens, Janet Strachan, Tony Korman, Kristy Horan, Diane Daniel, Madeline Russell, Christopher A. McDevitt, Norelle L. Sherry, Timothy P. Stinear, Benjamin P. Howden, Claire L. Gorrie

**Affiliations:** 1 Department of Microbiology and Immunology, University of Melbourne, at the Peter Doherty Institute for Infection and Immunity, Melbourne, Victoria, Australia; 2 Microbiological Diagnostic Unit Public Health Laboratory, Department of Microbiology and Immunology, University of Melbourne, at the Peter Doherty Institute for Infection and Immunity, Melbourne, Victoria, Australia; 3 Communicable Diseases Branch, Department of Health, Victoria, Australia; 4 Department of Microbiology, Monash Health, Clayton, Victoria, Australia; 5 Department of Infectious Diseases, Austin Health, Heidelberg, Victoria, Australia; 6 Centre for Pathogen Genomics, University of Melbourne, Melbourne, Victoria, Australia; Johns Hopkins University School of Medicine, Baltimore, Maryland, USA

**Keywords:** *Streptococcus pneumoniae*, antibiotic resistance, genomics

## Abstract

*Streptococcus pneumoniae* is a major human pathogen with a high burden of disease. Non-invasive isolates (those found in non-sterile sites) are thought to be a key source of invasive isolates (those found in sterile sites) and a reservoir of anti-microbial resistance (AMR) determinants. Despite this, pneumococcal surveillance has almost exclusively focused on invasive isolates. We aimed to compare contemporaneous invasive and non-invasive isolate populations to understand how they interact and identify differences in AMR gene distribution. We used a combination of whole-genome sequencing and phenotypic anti-microbial susceptibility testing and a data set of invasive (*n* = 1,288) and non-invasive (*n* = 186) pneumococcal isolates, collected in Victoria, Australia, between 2018 and 2022. The non-invasive population had increased levels of antibiotic resistance to multiple classes of antibiotics including beta-lactam antibiotics penicillin and ceftriaxone. We identified genomic intersections between the invasive and non-invasive populations and no distinct phylogenetic clustering of the two populations. However, this analysis revealed sub-populations overrepresented in each population. The sub-populations that had high levels of AMR were overrepresented in the non-invasive population. We determined that WamR-Pneumo was the most accurate *in silico* tool for predicting resistance to the antibiotics tested. This tool was then used to assess the allelic diversity of the penicillin-binding protein genes, which acquire mutations leading to beta-lactam antibiotic resistance, and found that they were highly conserved (≥80% shared) between the two populations. These findings show the potential of non-invasive isolates to serve as reservoirs of AMR determinants.

## INTRODUCTION

*Streptococcus pneumoniae,* also known as the pneumococcus, is a predominantly community-acquired opportunistic pathogen that colonizes the mucosal surface of the human upper respiratory tract and is a potential cause of local and invasive diseases (1). Invasive isolates, those found at normally sterile sites such as the blood, cause invasive pneumococcal disease (IPD) with presentations including septicaemia, meningitis, and pneumonia. Non-invasive isolates, those found in non-sterile sites such as the respiratory tract, are often associated with asymptomatic colonization but can also cause local diseases such as bronchitis, otitis media, and sinusitis. Non-invasive isolates act as a key source of invasive isolates; however, pathogen surveillance has predominantly focused on invasive isolates (2). The last comprehensive report comparing the prevalence of anti-microbial resistance (AMR) in invasive and non-invasive *S. pneumoniae* populations in Australia was performed in 2005 (3), while data on invasive isolates are regularly published (4).

The virulence of *S. pneumoniae* is highly dependent on the polysaccharide capsule, encoded by the capsular polysaccharide (*cps*) locus, with some capsule types more frequently associated with disease (1). *S. pneumoniae* vaccination strategies targeting virulent and IPD-causing serotypes have achieved a reduction in the number of IPD cases. However, these numbers have risen in recent years and have begun to approach pre-vaccine levels (5). One contributing factor to this occurrence is the capacity of *S. pneumoniae* to undergo serotype switching, whereby the *cps* locus is altered through recombination, resulting in an alternate serotype that can enable vaccine evasion (6, 7).

Antibiotic resistance in *S. pneumoniae* is also a growing concern. Once universally susceptible to penicillin, the first penicillin-resistant *S. pneumoniae* was reported in Australia in 1967, and resistance to beta-lactam antibiotics has been steadily increasing since that time. Resistance to macrolides and tetracyclines antibiotics has also been documented in *S. pneumoniae* over the intervening years (8 – 10). The molecular basis of pneumococcal resistance to beta-lactam antibiotics in pneumococci is predominantly, but not exclusively, caused by mutations in three penicillin-binding proteins (*pbp*’s) (*pbp1a*, *pbp2b*, and *pbp2x*), two of which (*pbp1a* and *pbp2x*) are situated at either side of the *cps* locus (11 – 14). Recombination within these regions could therefore facilitate simultaneous evasion of both vaccines and antibiotic pressure in a single recombination event (15). In parallel, the acquisition of whole genes such as *ermB* and *tetM* confers resistance to macrolides and tetracycline, respectively (16).

The ability of *S. pneumoniae* to readily recombine genomes highlights the potential of invasive and non-invasive populations to interact and share genetic material. This necessitates the use of whole-genome sequencing (WGS), as the genes encoding phenotypic characteristics, such as serotype and antibiotic resistance, can differ greatly between isolates despite having very similar genomes overall. In addition to well-established genomic categorization methods such as multi-locus sequence type (MLST), global pneumococcal sequence cluster (GPSC), and *in silico* serotyping using seroBA (17, 18), WGS is increasingly being used to detect the presence of AMR genes and mutations. The variability in *pbp* allelic combinations that can confer resistance to beta-lactam antibiotics have made it difficult to predict using *in silico* tools. However, the expansion of databases containing both WGS and phenotypic data has opened the way to improvements in *in silico* resistance identification. A recent paper by Demczuk et al. (19) used linear regression to accurately predict beta-lactam antibiotic resistance via WGS in a defined subset of strains. This allows for an approach where isolates can be classified as resistant if they have an accumulation of mutations in key binding motifs, with each change increasing the minimum inhibitory concentration by a custom amount, rather than the traditional binary presence/absence approach. Use of these tools is also an effective method for understanding the genotypic mechanism of any observed differences in resistance phenotypes and relating any changes to serotype switching events.

In this study, we compared the population structure and AMR profiles of invasive and non-invasive pneumococcal isolates collected in Victoria, Australia, to understand how the two interact and gain insight into the spread of AMR genes. The non-invasive population had increased levels of antibiotic resistance to a range of beta-lactam antibiotics including penicillin and ceftriaxone. We identified significant overlaps in the two populations with some serotypes, sequence types (STs), and GPSCs being significantly overrepresented in each of the populations. We compared the accuracy of *in silico* AMR prediction tools and validated WamR-Pneumo for use in routine *S. pneumoniae* surveillance for public health. We then used this tool to assess the allelic diversity of the *pbp* genes and found they were highly conserved, with ≥80% of alleles of each *pbp* shared between the two populations.

## MATERIALS AND METHODS

### Study design and data set

#### Invasive pneumococcal isolates

Pneumococcal isolates included in this study were collected from cases of IPD (isolates found at normally sterile sites such as the bloodstream) between August 2018 and December 2021 (inclusive) as part of the state laboratory-based surveillance in Victoria, Australia [population 6.5 million in 2021 (20)]. All invasive pneumococcal disease cases are required by legislation to be notified to the state health department, and isolates sent to the state public health laboratory for characterization. An invasive pneumococcal disease case was defined as pneumococci recovered from a normally sterile site. Following exclusion of isolates due to loss of samples and missing data, we included a total of 1,288 IPD isolates in this study; 298 from 2018, 428 from 2019, 172 from 2020, 220 from 2021, and 170 from 2022. Most samples were isolated from blood (96%) with the remaining samples from cerebrospinal fluid, lung fluid, joints, or other.

#### Non-invasive isolates

Non-invasive isolates (those found in non-sterile sites such as the respiratory tract, can be disease causing or non-disease-causing carriage) included here were collected in two studies. The first involved collection from a single large hospital network in Melbourne, Australia, from March 2021 to March 2022 (*n* = 69). Non-duplicate isolates (exclude duplicates within 14 days) of non-invasive *S. pneumoniae* from inpatients and outpatients were included. The second study was a state-wide AMR snapshot, collecting all non-invasive *S. pneumoniae* isolates from all Victorian diagnostic microbiology laboratories over a 1-month period in June 2022 (*n* = 117).

### Anti-microbial susceptibility testing

Anti-microbial susceptibility testing was performed using Sensititre broth microdilution on STP6F plates (Thermo Fisher Scientific, Waltham, MA, USA). A 0.5-McFarland suspension of bacterial isolates from overnight cultures (HBA at 36°C in CO_2_). Sensititre plates were inoculated with the bacterial suspensions and horse blood broth (Thermo Fisher Scientific) according to manufacturer’s instructions, incubated for 24 h at 36°C in CO_2_, and read using the fluorescence-based OptiRead system (Thermo Fisher Scientific). The MIC results were interpreted and categorized according to Clinical and Laboratory Standards Institute breakpoints 2021 (21).

### Whole-genome sequencing

Genomic DNA was extracted from bacterial isolates using a JANUS automated workstation (PerkinElmer) and Chemagic magnetic bead technology (PerkinElmer). Genomic DNA libraries were then prepared using the Nextera XT kit according to the manufacturer’s instructions (Illumina Inc.). Whole-genome sequencing was performed on the Illumina NextSeq platform using 2 × 150 bp paired-end chemistry.

### Genome quality control, assembly, and mapping

The Bohra (https://github.com/kristyhoran/bohra) pipeline was used to conduct quality control and to assemble the isolate genomes. Reads underwent quality control (default thresholds); genomes were assembled using SKESA (22) (v.2.4.0) within the shovill (https://github.com/tseemann/shovill) wrapper (v.1.0.4) using default parameters. All core genome alignments were performed using snippy (v.4.4.5, https://github.com/tseemann/snippy) with default parameters (minfrac = 0.9 and mincov = 10). The reference used for all analyses was ASM966447v1 (GCA_009664475.1), a locally collected Australian isolate (collected 2014, serotype 19A, ST199).

### Typing

Serotyping was performed *in silico* using seroBA (v.1.0.2) (17) for all isolates. MLST was performed using mlst (v.2.19.0) (https://github.com/tseemann/mlst) and the pubMLST database (https://pubmlst.org/) (23) for *S. pneumoniae*. A GPSC was assigned for all isolates using a *k*-mer-based clustering method PopPUNK (v.2.4) (24) and using the pneumococcal isolates in the Global Pneumococcal Sequencing (GPS) database (GPSC v.6) (https://www.pneumogen.net/gps/training_command_line.html) (18) as a reference. Minor types/clusters were defined as having <10 isolates in either of the invasive or non-invasive populations over the entire study period. Differences in the proportion of each type/cluster were assessed using a Proportion Test.

### *In silico* anti-microbial resistance gene detection

*In silico* prediction of anti-microbial susceptibility was performed using three methods (Ariba, abritAMR, and WamR-Pneumo), each of which uses an independent AMR gene database (CARD, NCBI AMRFinder-Plus, and a custom database, respectively). To allow for accurate comparisons between the three methods, a subset of the clinically relevant and mechanistically well-studied antibiotic susceptibility phenotypes were selected, including penicillin (oral breakpoints), ceftriaxone (meningitis breakpoints), cefuroxime, erythromycin, levofloxacin, and tetracycline. An overview of this process can be found in Fig. S1.

#### Method 1

abritAMR (25) utilizes the NCBI AMRFinderPlus database (26) for detection of AMR genes. This tool is used as part of the routine public health genomics pipeline for state-wide surveillance of *S. pneumoniae* in Victoria. Analysis was performed on isolate assemblies using default settings and the species flag (--species *Streptococcus pneumoniae*). Mutational resistance has not been validated and implemented for *S. pneumoniae* using this tool. This tool assess if there are known susceptible *pbp* alleles present in a sample and will flag isolates that do not have a known susceptible allele. In this way, it is not labeling isolates as resistant; rather, it is labeling them as most likely not susceptible.

#### Method 2

WamR-Pneumo (https://github.com/phac-nml/wade) utilizes a custom database provided by the tool. The AMR gene detection strategy and MIC prediction logic used in the tool is outlined by Demczuk et al. (19). To provide an allele identification number, WamR-Pneumo was run twice: first, to identify any novel AMR alleles, which were then numbered and added to the database; and then a second time to assign known and newly identified alleles. The algorithms used to determine the genotypically inferred MIC for beta-lactam antibiotics are provided in Fig. S2.

#### Method 3

ARIBA (v.2.14.6) (27) utilizes the CARD database (https://card.mcmaster.ca/home) for detection of AMR genes. The default settings were used with isolate reads as the input. Data in the CARD database were then used to determine which drug class the variant/gene conferred resistance to.

For each method tested, the proportion of major and minor errors was then determined. Major errors were defined as isolates that were phenotypically resistant or intermediate but labeled as susceptible by genotype. Minor errors were defined as isolates that were phenotypically susceptible but labeled as resistant/intermediate by genotype.

### Phylogenetic trees

Phylogenetic trees were constructed using whole-genome alignments and inferred using IQtree (28) (v.2.1.4) with constant sites, 1,000 bootstraps, and a generalized time-reversible model of evolution (GTR + G4). The reference used for all whole-genome alignment phylogenies was ASM966447v1 (accession number: GCA_009664475; ST199, serotype 19A, 2,090,792 bp). For the species alignment, the core alignment length was 1,320,584 bp, and the core single nucleotide polymorphism (SNP) alignment was 69,774 sites. Recombination masking was not performed on the species whole-genome alignment due to the small size of the resulting alignment.

For phylogenies using protein alignments, unique proteins were identified using cd-hit-dup (v.4.8.1) (29, 30), and these were then aligned using muscle (v.3.8.1551) (31) with default settings. IQtree (v.2.1.4) was used to build the phylogenies with 1,000 bootstraps and using the LG (general matrix) amino acid exchange rate matrix (32).

All trees were midpoint rooted and visualized in R (v.4.1.2, https://www.R-project.org/) using a combination of phangorn (33), ggtree (34), ggnewscale (https://eliocamp.github.io/ggnewscale/), ggmsa (35), and aplot (https://github.com/YuLab-SMU/aplot).

### Data visualization and statistics

Figures were generated in R (v.4.1.2) (https://www.R-project.org/) using the tidyverse suite (36) and rstatix (https://github.com/kassambara/rstatix) to compute statistics. To assess any differences in the proportion of isolates that were resistant and multi-drug resistant, a proportion test (two-sided) was performed. To assess any differences in the anti-microbial susceptibility profiles of the invasive and non-invasive populations, Fisher’s exact test was conducted for each antibiotic, comparing the proportion of each resistance classification. To assess differences in the proportions of types/clusters in the invasive and non-invasive populations, a proportion test (two-sided) was performed on each type/cluster.

## RESULTS

### The non-invasive isolates had significantly higher levels of AMR

All isolates underwent extensive phenotypic susceptibility testing using the broth microdilution method. However, overall levels of resistance in both populations were low, and the two populations did not have significantly different levels of resistance based on the number of isolates resistant to at least one class of antibiotics (Table S1). In the invasive population, more than 10% resistance was observed for penicillin (using meningitis breakpoints, 20.3%), azithromycin (10.8%), erythromycin (11.3%), tetracycline (10.5%), and trimethoprim (12.5%). In the non-invasive population, more than 10% resistance was observed in penicillin (using meningitis breakpoints, 31.4%), cefuroxime (12.4%), azithromycin (14.6%), clindamycin (10.3%), erythromycin (14.6%), tetracycline (13%), and trimethoprim (20.5%) (Table 1).

**TABLE 1:** Anti-microbial susceptibility results of *S. pneumoniae* population^*a*^

Antibiotic sub-class	Antibiotic (men/non-men breakpoint used, if applicable)	Population	Total number of isolates tested	Number of sensitive isolates (%)	Number of intermediate isolates (%)	Number of resistant isolates (%)	*P* value (Fisher’s exact test)
Beta-lactam	Amox-clav acid	Invasive	1,176	1,145(97.4)	17(1.4)	14(1.2)	0.13074
		Non-invasive	169	161(95.3)	3(1.8)	5(3%)	
	Cefepime (men)	Invasive	**1,286**	**1,205 (93.7)**	**41 (3.2)**	**40 (3.1)**	0.00581*
		Non-invasive	**185**	**162 (87.6)**	**15 (8.1)**	**8 (4.3)**	
	Cefepime (non-men)	Invasive	1,286	1,246(96.9)	36(2.8)	4(0.3)	0.38907
		Non-invasive	185	177(95.7)	8(4.3)	0(0)	
	Cefotaxime (men)	Invasive	**1,286**	**1,218 (94.7)**	**44 (3.4)**	**24 (1.9)**	0.02765*
		Non-invasive	**185**	**167 (90.3)**	**14 (7.6)**	**4 (2.2)**	
	Cefotaxime (non-men)	Invasive	1,286	1,262(98.1)	24(1.9)	0(0)	0.77197
		Non-invasive	185	181(97.8)	4(2.2%)	0(0)	
	Ceftriaxone (men)	Invasive	**1,285**	**1,200 (93.4)**	**46 (3.6)**	**39 (3)**	0.00977*
		Non-invasive	**185**	**161 (87)**	**14 (7.6)**	**10 (5.4)**	
	Ceftriaxone (non-men)	Invasive	1,285	1,246(97)	35(2.7)	4(0.3)	0.15429
		Non-invasive	185	175(94.6)	10(5.4)	0(0)	
	Cefuroxime	Invasive	**1,286**	**1,190 (92.5)**	**19 (1.5)**	**77 (6)**	0.00621*
		Non-invasive	**185**	**159 (85.9)**	**3 (1.6)**	**23 (12.4)**	
	Ertapenem	Invasive	1,286	1,278(99.4)	8(0.6)	0(0)	0.36423
		Non-invasive	185	183(98.9)	2(1.1)	0(0)	
	Meropenem	Invasive	**1,286**	**1,220 (94.9)**	**44 (3.4)**	**22 (1.7)**	0.01779*
		Non-invasive	**185**	**166 (89.7)**	**12 (6.5)**	**7 (3.8)**	
	Penicillin (men)	Invasive	**1,285**	**1,024 (79.7)**	**0 (0)**	**261 (20.3)**	0.00111*
		Non-invasive	**185**	**127 (68.6)**	**0 (0)**	**58 (31.4)**	
	Penicillin (non-men)	Invasive	1,285	1,251(97.4)	30(2.3)	4(0.3)	0.23032
		Non-invasive	185	177(95.7)	8(4.3)	0(0)	
	Penicillin (oral)	Invasive	**1,285**	**1,024 (79.7)**	**196 (15.3)**	**65 (5.1)**	0.0021*
		Non-invasive	**185**	**127 (68.6)**	**40 (21.6)**	**18 (9.7)**	
Chloramphenicol	Chloramphenicol	Invasive	1,286	1,265(98.4)	NA	21(1.6)	0.54424
		Non-invasive	185	181(97.8)	NA	4(2.2)	
Glycopeptide	Vancomycin	Invasive	1286	1,286(100)	NA	NA	1
		Non-invasive	185	185(100)	NA	NA	
Macrolide	Azithromycin	Invasive	1,135	1,012(89.2)	0(0)	123(10.8)	0.14868
		Non-invasive	164	140(85.4)	NA	24(14.6)	
	Clindamycin	Invasive	1,286	1,172(91.1)	8(0.6)	106(8.2)	0.50265
		Non-invasive	185	166(89.7)	NA	19(10.3)	
	Erythromycin	Invasive	1,286	1,133(88.1)	8(0.6)	145(11.3)	0.34623
		Non-invasive	185	157(84.9)	1(0.5)	27(14.6)	
Oxazolidinone	Linezolid	Invasive	1,286	1,286(100)	NA	NA	1
		Non-invasive	185	185(100)	NA	NA	
Quinolone	Levofloxacin	Invasive	1,285	1,281(99.7)	2(0.2)	2(0.2)	1
		Non-invasive	185	185(100)	0(0)	0(0)	
	Moxifloxacin	Invasive	1,285	1,280(99.6)	4(0.3)	1(0.1)	1
		Non-invasive	185	185(100)	0(0)	0(0)	
Tetracycline	Tetracycline	Invasive	1,286	1,129(87.8)	22(1.7)	135(10.5)	0.35615
		Non-invasive	185	160(86.5)	1(0.5)	24(13)	
Trimethoprim	Trimeth-sulfa	Invasive	**1,286**	**1,003 (78)**	**122 (9.5)**	**161 (12.5)**	0.01271*
		Non-invasive	**185**	**134 (72.4)**	**13 (7)**	**38 (20.5)**	

^*a*^ The population has been separated based on invasive (*n* = 1,286) or non-invasive (*n* = 185). Amox-clav acid refers to amoxicillin and clavulanic acid, and trimeth-sulfa refers to trimethoprim-sulfamethoxazole. NA rows indicate that there is no such breakpoint for that antibiotic. Breakpoints are based on the 2022 CLSI guidelines. Men refers to meningitis breakpoints; non-men refers to non-meningitis breakpoints; and oral refers to oral breakpoints. *P* values were calculated using Fisher’s exact test, and values less than 0.05 are indicated with an * and are bolded.

The resistance profiles of the two populations were then compared to determine if either had an increased proportion of non-susceptible isolates (i.e., intermediate and resistant). The non-invasive population had significantly increased resistance (*P* < 0.05) to the antibiotics cefepime (using meningitis breakpoints), cefotaxime (using meningitis breakpoints), ceftriaxone (using meningitis breakpoints), cefuroxime, meropenem, penicillin (using meningitis and oral breakpoints), and trimethoprim compared to the invasive population. The data showed that the invasive population did not have statistically increased resistance to any antibiotics (Table 1), by comparison to the non-invasive population.

When investigating the levels of multi-drug resistance (defined as resistance to three or more antibiotic classes) in the two populations, we observed that the proportion of multi-drug resistant isolates was significantly increased in the non-invasive population (10.2%) compared to the invasive isolates (4.6%). In addition, the proportion of isolates that were resistant to one anti-microbial class that were multi-drug resistant was significantly higher in the non-invasive compared to the invasive population (35.2% compared to 19.2%, *P* < 0.01) (Table S1). The cooccurrence of resistance to each microbial class was similar between the two populations (Fig. S2).

### The invasive and non-invasive population structures were similar but with notable exceptions

**Fig 1:**
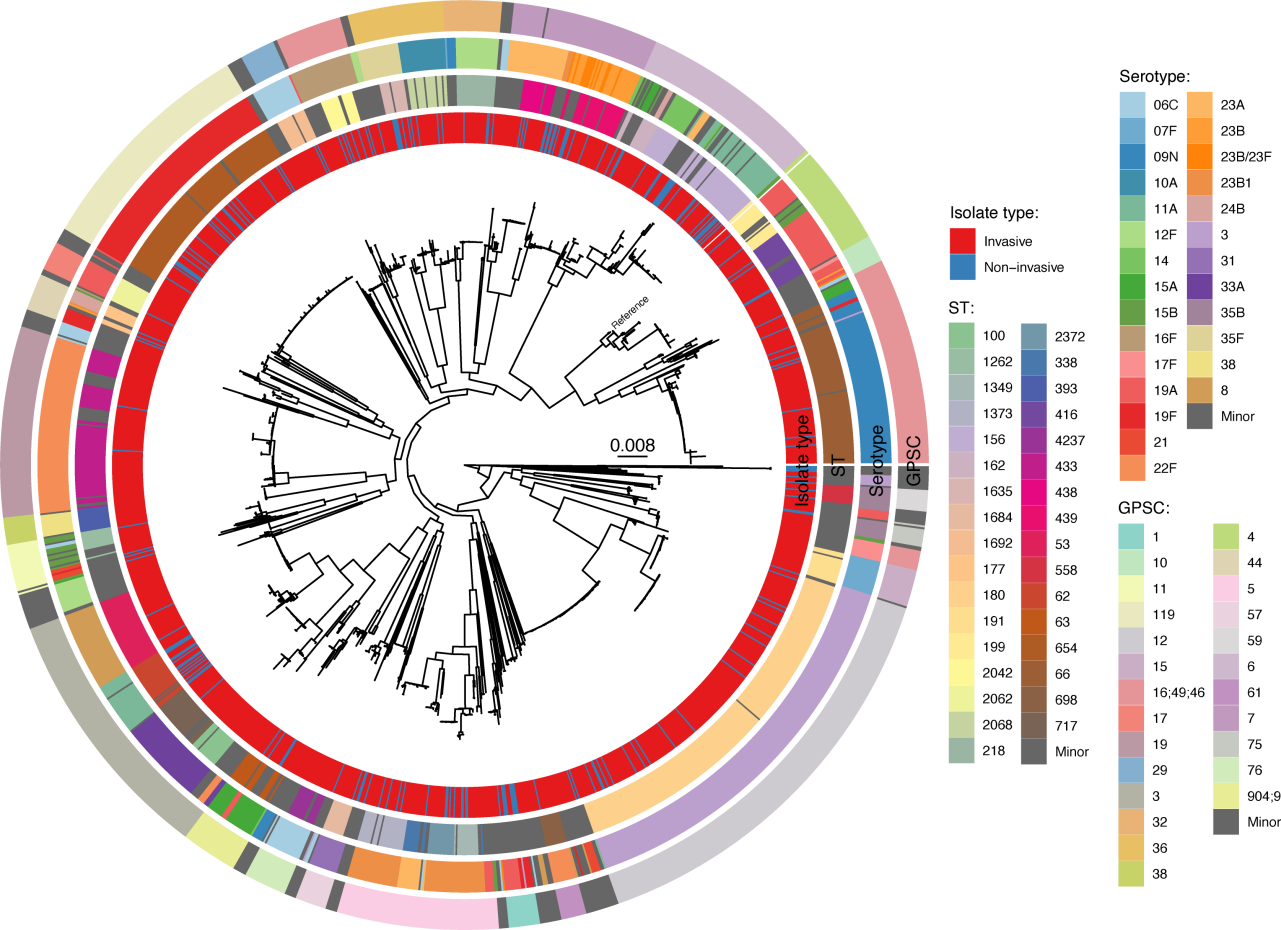
Midpoint-rooted maximum likelihood phylogenetic tree of all study isolates (*n* = 1,474). Minor serotypes, sequence types (STs), and global pneumococcal sequence cluster (GPSC) were defined as those that contained less than 10 isolates over the study period. The reference was ASM966447v1 (GCA_009664475.1), collected 2014, serotype 19A, ST199.

After determining that the non-invasive population had increased levels of anti-microbial resistance compared to the invasive population, we investigated if this was due to the presence of subpopulations unique to the non-invasive population. Accordingly, a phylogenetic tree was generated to provide deeper insight into the genomic relationships between the invasive and non-invasive population groups. This showed extensive overlap between the genomic population structures of the two groups, with most serotypes, STs, and GPSCs found in both populations. No distinct clustering of non-invasive isolates beyond that which was associated with ST, serotype, or GPSC was observed (Fig. 1).

**Fig 2:**
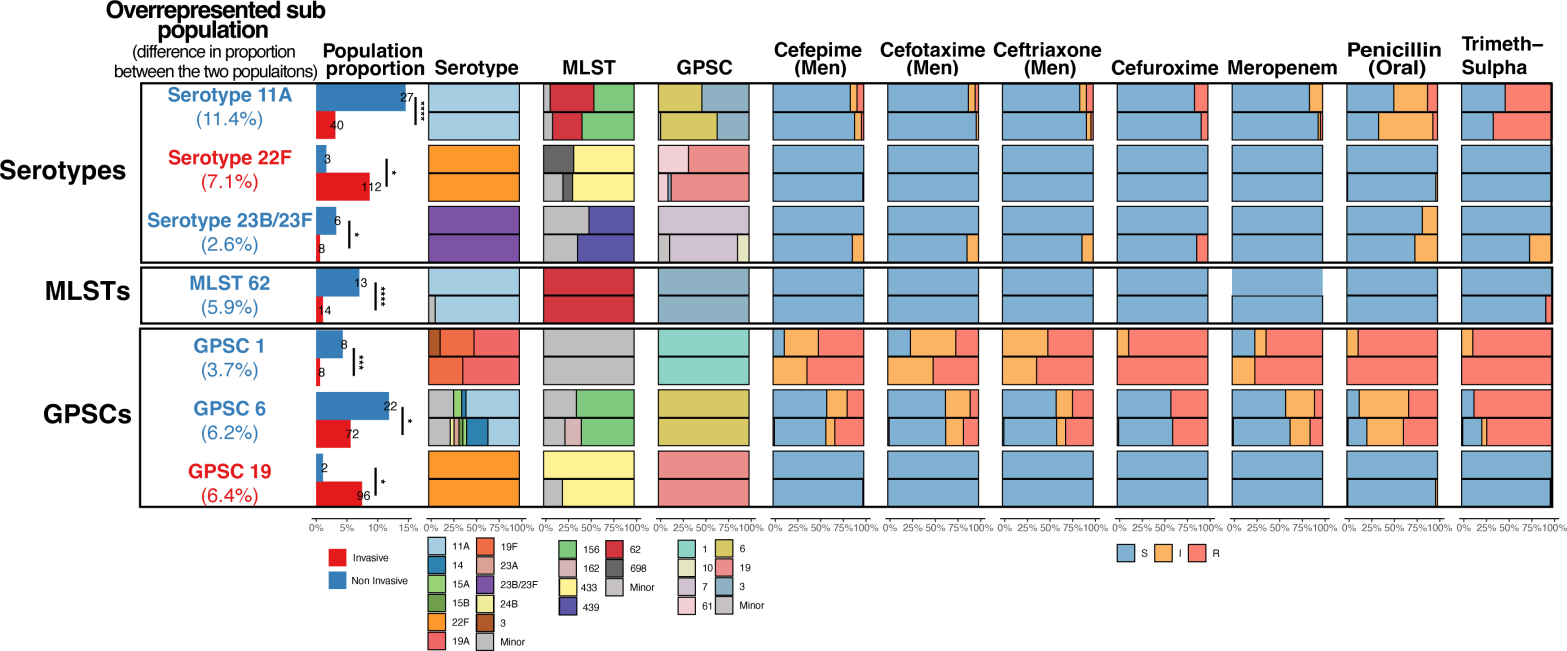
Summary of the key characteristics of the overrepresented sub-populations. The antibiotics included in the diagram are those that had a significant difference between the two populations (as per Table 1). Trimeth-sulfa refers to trimethoprim-sulfamethoxazole. The overrepresented sub-populations are colored based on which data set had the larger proportion of isolates: red for invasive and blue for non-invasive. The percentages displayed under the overrepresented population type/clusters are the differences in proportion between the two populations. A count of isolates for each typing method is displayed at the top of the bar chart. The proportion of isolates that were susceptible (blue), intermediate (orange), or resistant (red) to the antibiotics listed is also displayed [CLSI (21) breakpoints, meningitis/oral breakpoints used where indicated]. Minor types/clusters are defined as those having <10 isolates over the whole study period.

However, there were exceptions, with seven types/clusters significantly overrepresented in either population (*P* < 0.05). This included serotypes 11A (11.4% more in non-invasive, 22F (7.1% more in invasive) and 23B/23F (2.6% more in non-invasive), ST 62 (4.3% more in non-invasive), and GPSCs 1 (3.7% more in non-invasive), 19 (6.4% more in invasive), and 6 (6.2% more in non-invasive) (Fig. 2; Fig. S3).

The characteristics of the significantly overrepresented types/clusters were investigated further to determine if there were differences between the two populations. The overrepresented types/clusters had similar genomic diversity and rates of resistance in the invasive and non-invasive populations. However, the types/clusters overrepresented in the non-invasive population often had a high rate of resistance (>20% isolates resistant to at least one antibiotic that we observed significant differences between the invasive and non-invasive populations). This included serotypes 11A and 23B1, ST 156, and GPSCs 1 and 6. In contrast, the types/clusters overrepresented in the invasive population, such as serotype 22F and GPSC 19, had very low rates of resistance (Fig. 2).

There was an overlap between the overrepresented types/clusters but not across all typing/clustering schemes. Although all GPSC 19 isolates were serotype 22F, not all serotype 22F isolates were GPSC 19, with 14% belonging to GPSCs 61 (*n* = 13) and 3 (*n* = 3). Of the GPSCs that contributed to the differences in levels of resistance between the two populations, GPSC 1 consisted exclusively of minor STs with all but one isolate being serotype 19A or 19F. Isolates belonging to GPSC 6 were more diverse and represented a range of serotypes and STs, predominantly being serotype 11A and ST 156 (Fig. 2; Fig. S4).

### WamR-Pneumo was the best-performing *in silico* resistance prediction tool

To investigate the diversity of the genes encoding resistance, we first compared *in silico* resistance prediction tools to phenotypic AST results to understand their accuracy in AMR phenotype prediction.

**Fig 3:**
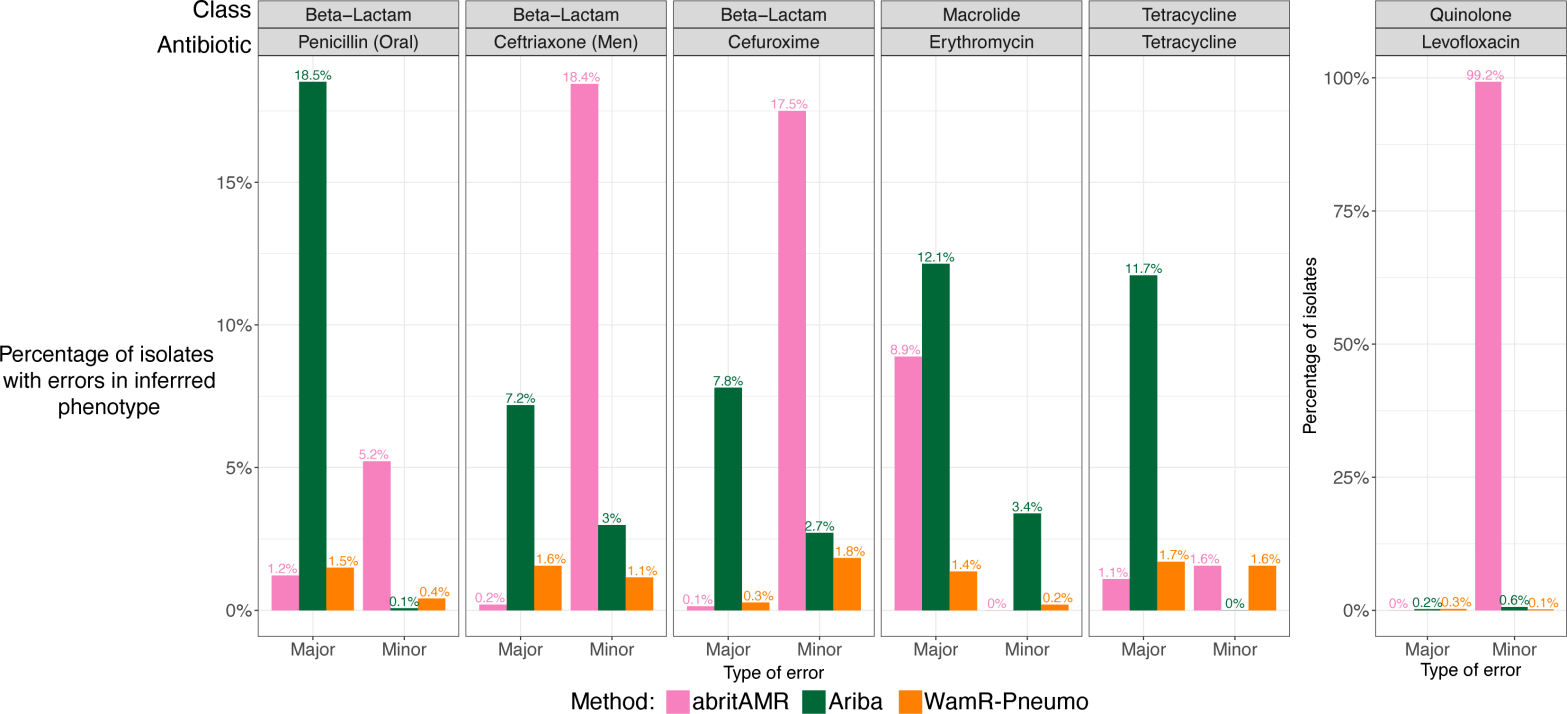
Validation of the *in silico* resistance prediction method. Proportion of isolates classified as major or minor errors for each *in silico* resistance prediction method. Major errors were defined as isolates that were phenotypically resistant or intermediate but labeled as susceptible by genotype. Minor errors were defined as isolates that were phenotypically susceptible but labeled as resistant/intermediate by genotype. Isolates phenotypically classified as intermediate or resistant were grouped to allow uniform comparison between all the methods.

The best-performing *in silico* resistance prediction tool was WamR-Pneumo, with consistently low rates of major and minor errors for all antibiotics investigated. Though Ariba and abritAMR did have lower rates of major or minor errors than WamR-Pneumo for some antibiotics, such as ceftriaxone and tetracycline, these methods often had the low rates in one type of error at the cost of having a much higher rate in the other error category, for example, abritAMR with ceftriaxone (Fig. 3).

WamR-Pneumo is also able to predict a phenotypic MIC based on the genomic data (genotypic MIC). The inferred genotypic MIC was compared to the phenotypic MIC to understand the number and magnitude of the errors, defined as the number of dilutions different between genotypic and phenotypic MIC values. For genotypic MICs that had the same resistance interpretation as phenotypic MICs, most antibiotics (six out of eight) were very accurately predicted, with most isolates having no dilutions differing between the genotypic and phenotypic MICs. In contrast, macrolide antibiotics azithromycin and erythromycin had genotypic MICs that were consistently one dilution less than phenotypic MICs. For isolates where the differences in genotypic and phenotypic MICs resulted in a difference in the MIC resistance classification, these were most common for beta-lactam antibiotics, tetracycline, and trimethoprim-sulfamethoxazole. However, these isolates represented a small proportion of the total population (Fig. S5).

### Beta-lactam antibiotic resistance mechanisms are highly conserved between the invasive and non-invasive populations

Following identification of WamR-Pneumo as the most accurate *in silico* resistance prediction tool, the allelic diversity of the three *pbp* genes, which are a primary driver of resistance *S. pneumoniae* to beta-lactam antibiotics, was assessed to determine the extent of overlap between the invasive and non-invasive populations. The analysis focused on beta-lactam antibiotics as these are clinically relevant antibiotics to which the non-invasive population has been shown to have increased resistance.

**Fig 4:**
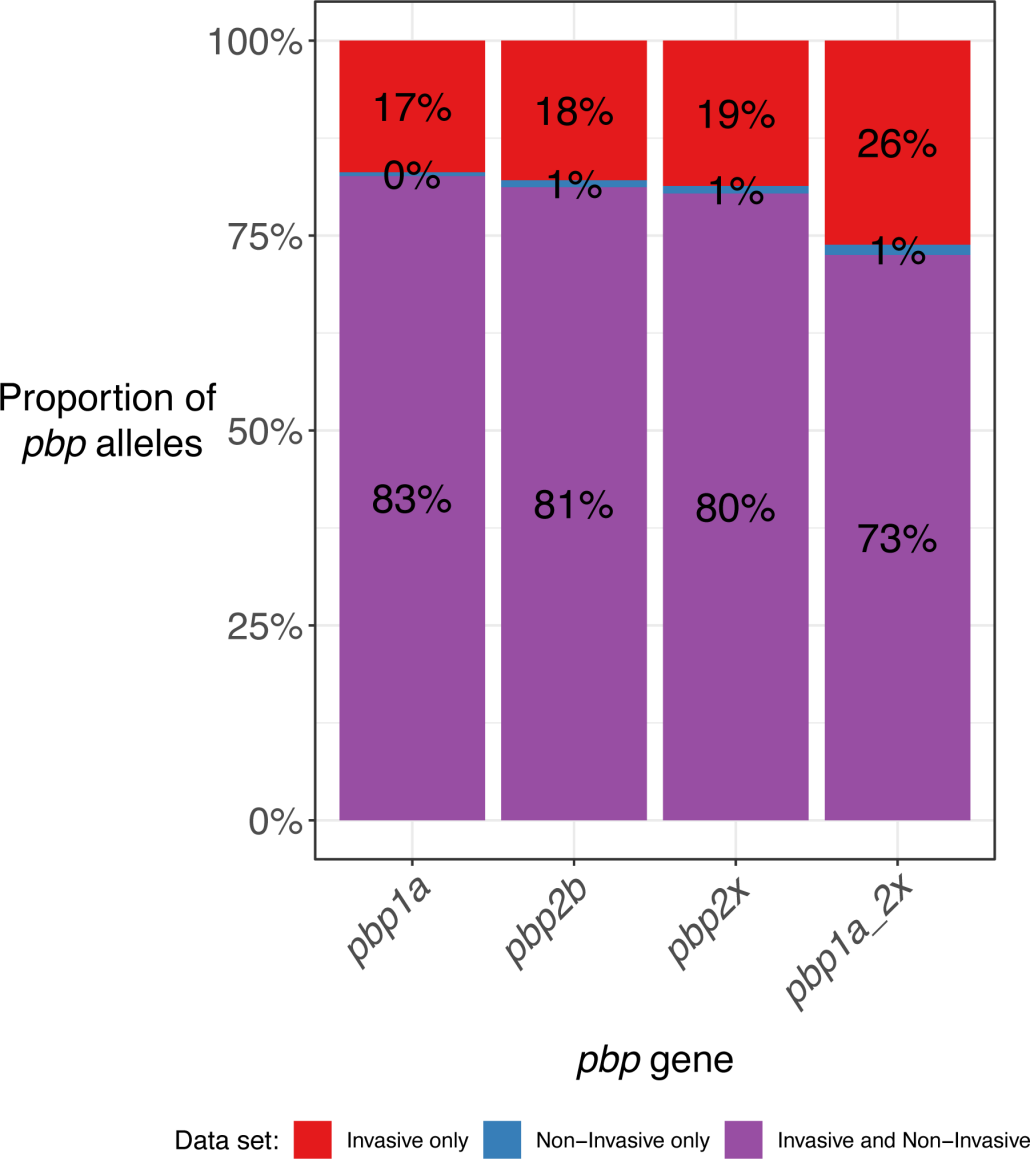
Proportion of *pbp* alleles that were unique to each data set. The category *pbp1a_2x* is based on a combined *pbp1a* and *pbp2x* allele.

The diversity of the *pbp*s was extensive. Of the three genes, *pbp2x* was the most diverse, with 173 unique alleles identified, followed by *pbp2b* with 155 unique alleles and *pbp1a* with 127 unique alleles. The diversity of the corresponding protein sequences was lower, with PBP2x, PBP2b, and PBP1a having 126, 102, and 78 unique sequences, respectively (Table S2). The majority (>80%) of the unique *pbp* alleles identified for each gene were shared between the invasive and non-invasive populations. The extent of sharing of the combination of *pbp1a* and *pbp2x* alleles was also investigated as these two genes, along with the *cps* locus that encodes the serotype, can be collectively exchanged in a single recombination event. This revealed that specific combinations of the *pbp1a* and *pbp2x* alleles were shared at similar proportions to the individual genes (Fig. 4).

**Fig 5:**
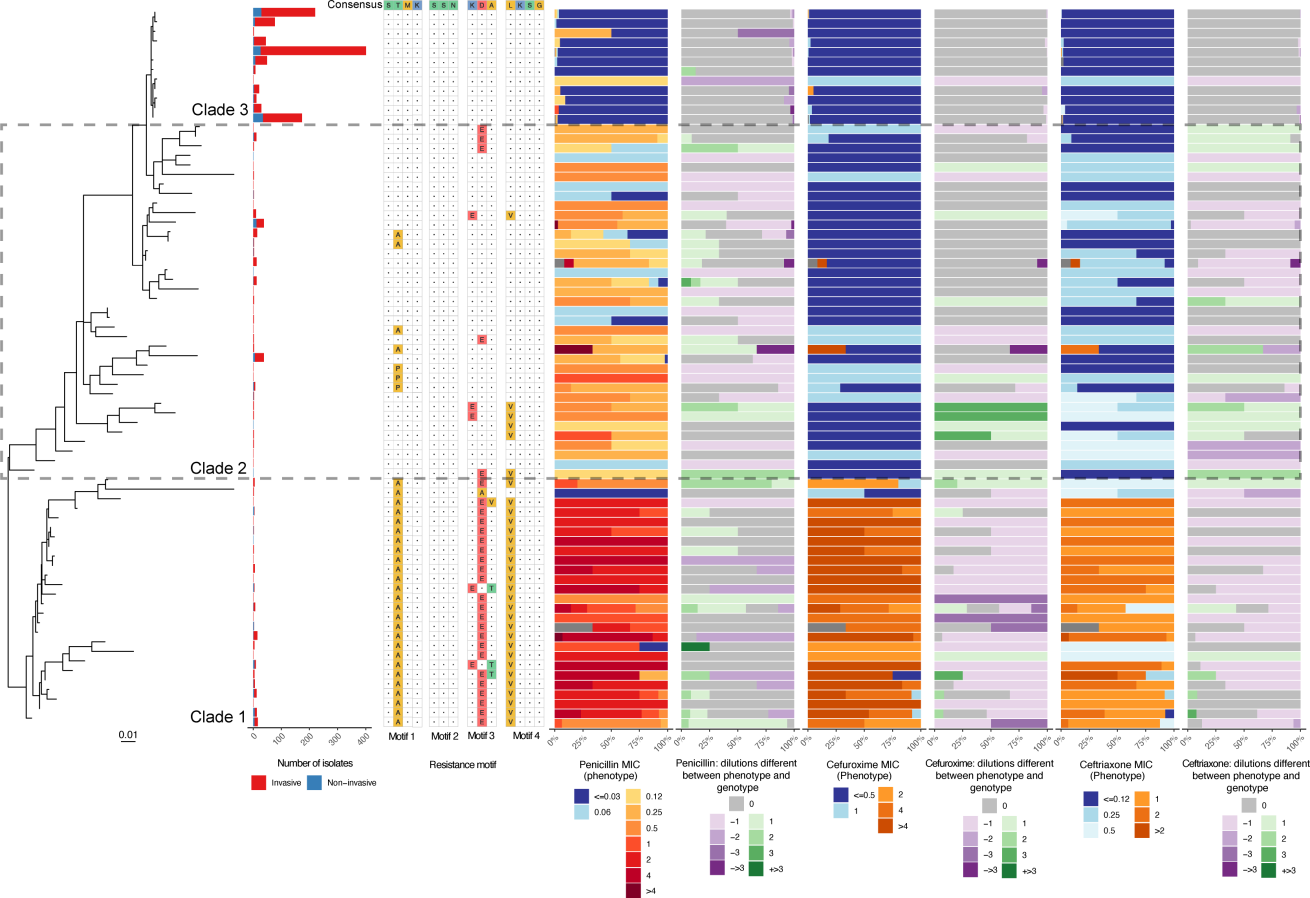
Midpoint rooted maximum likelihood phylogenetic tree of PBP2x. Tree has been built using an amino acid sequence alignment of all unique proteins that had at least one isolate with at least one dilution difference between the phenotypic and genotypic MICs. The number of isolates is based on counts of isolates that had contained the PBP2x alleles. The resistance motifs are the motifs used by WamR-Pneumo to determine the beta-lactam MIC, and the amino acids have been colored based on their side-chain chemistry. The MIC panels [penicillin (oral breakpoints), cefuroxime (meningitis breakpoints) and ceftriaxone (meningitis breakpoints)] show the proportion of isolates with each MIC for each of the PBP2x alleles (blue for susceptible and orange/red for intermediate/resistant). The panels displaying the number of dilutions different between the phenotype and the genotype used WamR-Pneumo to infer the MIC from the genotype.

A phylogenetic tree was constructed for each unique PBP protein sequence that had at least one isolate with discrepant (≥1 dilutions different) phenotypic and genotypic MICs to understand the extent of allelic diversity and identify any putative novel resistance-encoding alleles. Consistent with expectations, there was a clear phylogenetic relationship with the phenotypic MIC for all beta-lactam antibiotics and changes in the four key resistance motifs (Fig. S6 to S9). This was true for PBP1a and PBP2b, but a notable exception was PBP2x. The PBP2x tree can be divided into three clades (Fig. 5), with clade 1 containing sequences with changes in at least two of the motifs and a strong association with high phenotypic MICs for all beta-lactam antibiotics. Clade 2 contained isolates that were consistently resistant to penicillin but did not identify consistent changes in the four key motifs known to encode resistance. This indicates the presence of undetected resistance mechanisms within this clade. Clade 3 alleles were from isolates that were consistently susceptible to all beta-lactam antibiotics investigated and had no changes in the four resistance motifs. All isolate counts for PBP2x sequences in clades 1 and 2 were less than 50, with most isolates in clade 3 (>70%) (Fig. 5).

## DISCUSSION

Rates of AMR for *S. pneumoniae* have been increasing globally, and it features on the World Health Organization and US Centers for Disease Control and Prevention priority pathogen lists (37, 38). This work establishes that the non-invasive population had significantly increased levels of resistance to beta-lactam antibiotics including penicillin, ceftriaxone, and cefuroxime. The observed differences in resistance levels between the two populations may also be associated with changes in virulence as evolution of resistance has been associated with a reduction in virulence (39, 40). The types/clusters that contributed most to the increased resistance in the non-invasive data set were GPSCs 1 and 6. GPSCs 1 and 6 have been shown internationally to have a high rate of AMR and multi-drug resistance (41), consistent with our study. Although some of the antibiotics identified as having significantly different resistance levels between the two populations are not clinically relevant, such as trimethoprim-sulfamethoxazole, these changes may reflect exposure to xenobiotic selective pressures encountered within community settings.

The overrepresented population with the highest levels of resistance was GPSC 1. Interestingly this cluster also had a high proportion of serotype 19A and 19F isolates, both of which are contained in the 13v-PCV. All GPSC 1 isolates, both invasive and non-invasive, were minor STs (<10 isolates in the study period). This suggests that these isolates could have been the product of a recombination event, resulting in strains that are better adapted to invasive infections as they are extensively drug resistant. This heterogeneity in ST is consistent with previous studies that have suggested that the continued prevalence of serotype 19 isolates, despite it being contained in the vaccine, is due to its diversity (42, 43).

As shown, the proportion of unique *pbp* alleles in each data set (invasive only or non-invasive only) was insufficient to explain the increased number of resistant isolates within the non-invasive population. This suggests that it was the overrepresented populations rather than a specific resistance mechanism that was responsible for the increased levels of resistance in the non-invasive population. Both *pbp1a* and *pbp2x* were examined in combination due to their colocation and proximity to the *cps* locus as well as their large contribution to determining resistance to beta-lactam antibiotics. The small proportion of *pbp1a-pbp2x* combination alleles unique to the non-invasive population further reinforced that the difference in resistance levels was not due to unique resistance alleles within a single population.

The PBP2x alleles in clade 2 had decreased susceptibility to penicillin, despite not having any changes in the recognized resistance motifs (Fig. 5). We do note that these alleles were uncommon in the total data set, with most isolates containing susceptible alleles that had no changes in any of the resistance motifs (clade 3). It remains unclear if the clade 2 isolates have reduced susceptibility due to changes in the other PBPs (PBP1a or PBP2b) or if there are unrecognized changes in PBP2x that are responsible. Additionally changes in other proteins such as heat shock protein ClpL have been shown to decrease penicillin susceptibility (44). However, other genes, such as *clpL*, were not assessed in our study as they are not contained in any of the databases currently used by the *in silico* prediction tools.

The high accuracy of WamR-Pneumo in predicting MICs could remove the need to perform labour-intensive phenotypic testing and better leverage existing WGS data to provide a more comprehensive understanding of the *S. pneumoniae* population beyond serotype and ST. Implementation of such a tool would likely require periodic phenotypic testing of a subset of isolates to ensure that tools remained accurate, and any novel variants were identified. When assessing the accuracy of the *in silico* resistance prediction tools, we aimed to use them as “out of the box” as possible to mimic how they would be potentially used by a public health surveillance laboratory. We acknowledge that the differences in major/minor error rates may be partially explained by the different databases used or the logic used to weight the impact of genes/mutations; however, this was not actively assessed during our study.

Our study also has implications for the implementation of genomics into routine surveillance and diagnostics. This is the first study of its kind conducted in Australia that compares the genotypic and phenotypic profiles of invasive and non-invasive isolates and shows the importance of monitoring both to ensure treatment decisions and guidelines remain appropriate. We have validated an *in silico* analysis method that can be readily implemented, allowing for accurate AMR phenotype prediction but also aiding in monitoring of AMR development and gene sharing. Studies such as ours are important in developing a surveillance baseline that future population changes can be measured against.

Although the non-invasive data set was collected from non-sterile sites, and these patients were not diagnosed with an invasive infection, we did not have additional information on the patient’s condition or treatment. These patients may have been receiving antibiotic treatment for an unrelated condition which selected for resistant pneumococcal strains. Therefore, it is possible that our isolate data set may have an elevated level of resistance due to the nature of the sampling, although this inference is speculative. It is also important to note that population complexity also arises from interactions between vaccination/vaccine serotypes, antibiotic pressure, and different disease types (i.e., carriage, non-invasive disease, and invasive disease), which are challenging factors to address in the context of studies such as this one.

Despite these potential limitations, we found strong evidence that shows the non-invasive *S. pneumoniae* population had increased resistance to a range of beta-lactam antibiotics as well as higher rates of multi-drug resistance. This increased resistance could be explained by an overrepresentation of AMR types/clusters, such as GPSCs 6 and 1 in the non-invasive population. We were able to show this using WGS and validating methods for *in silico* AMR prediction. Notably, although types/clusters were overrepresented in each population, we observed sharing of many AMR alleles between the two populations. Our results show the potential of a non-invasive population to act as a reservoir for AMR determinants in invasive infections and the utility of WGS in monitoring circulating AMR determinants and therefore the potential use of WGS in surveillance in public health. It is important to monitor both invasive and non-invasive populations for effective public health surveillance and ensuring clinical treatment guidelines remain appropriate. These data can be used to aid in decisions regarding changes to the serotype makeup of vaccines to assist with the management of AMR by targeting serotypes known to have high resistance rates.

## Data Availability

Genome sequences are deposited in GenBank under BioProject PRJNA857543, and the accession numbers and sample data are available in the supplementary material (data set S1). All supporting data, codes, and protocols have been provided within the article or through supplemental data files.

## SUPPLEMENTAL MATERIAL

Fig S1 aac.00785-23-s0001.pdf All supplemental figures.

Dataset S1 aac.00785-23-s0002.csv All metadata.

Dataset S2 aac.00785-23-s0003.csv Geno comp.

ASM does not own the copyrights to Supplemental Material that may be linked to, or accessed through, an article. The authors have granted ASM a non-exclusive, world-wide license to publish the Supplemental Material files. Please contact the corresponding author directly for reuse.

## References

[1] Weiser JN , Ferreira DM , Paton JC . 2018. Streptococcus pneumoniae: transmission, colonization and invasion. Nat Rev Microbiol 16:355–367. doi:10.1038/s41579-018-0001-8 29599457 PMC5949087

[2] Bogaert D , De Groot R , Hermans PWM . 2004. Streptococcus pneumoniae colonisation: the key to pneumococcal disease. Lancet Infect Dis 4:144–154. doi:10.1016/S1473-3099(04)00938-7 14998500

[3] Gottlieb T , Collignon PJ , Robson JM , Pearson JC , Bell JM , Australian Group on Antimicrobial Resistance . 2008. Prevalence of antimicrobial resistances in Streptococcus pneumoniae in Australia, 2005: report from the Australian group on antimicrobial resistance. Commun Dis Intell Q Rep 32:242–249. 18767424 10.33321/cdi.2008.32.23

[4] Australian Commission on Safety and Quality in Health Care. AURA 2021 Fourth Australian report on antimicrobial use and resistance in human health [Internet] . 2021. Available from: https://www.safetyandquality.gov.au/our-work/antimicrobial-resistance/antimicrobial-use-and-resistance-australia-surveillance-system/aura-2021

[5] Pennington K , Enhanced Invasive Pneumococcal Disease Surveillance Working Group, Communicable Diseases Network Australia . 2020. Invasive Pneumococcal disease surveillance, 1 July to 30 September 2019. Commun Dis Intell (2018) 44. doi:10.33321/cdi.2020.44.40 32418512

[6] Brueggemann AB , Pai R , Crook DW , Beall B . 2007. Vaccine escape recombinants emerge after pneumococcal vaccination in the United States. PLoS Pathog 3:e168. doi:10.1371/journal.ppat.0030168 18020702 PMC2077903

[7] Croucher NJ , Kagedan L , Thompson CM , Parkhill J , Bentley SD , Finkelstein JA , Lipsitch M , Hanage WP . 2015. Selective and genetic constraints on pneumococcal serotype switching. PLoS Genet 11:e1005095. doi:10.1371/journal.pgen.1005095 25826208 PMC4380333

[8] Fenoll A , Ardanuy C , Liñares J , Cercenado E , Marco F , Fleites A , Rodríguez-Mayo M , López-Hontangas J-L , Palop B , Aller A-I , Buendía B , Méndez C , Cifuentes I , ODIN Study Group . 2018. Serotypes and Genotypes of S. pneumoniae isolates from adult invasive disease in Spain: a 5-year prospective surveillance after pediatric Pcv13 licensure. The ODIN study. Vaccine 36:7993–8000. doi:10.1016/j.vaccine.2018.10.098 30449634

[9] Ruhe JJ , Myers L , Mushatt D , Hasbun R . 2004. High-level penicillin-nonsusceptible Streptococcus pneumoniae bacteremia: identification of a low-risk subgroup. Clin Infect Dis 38:508–514. doi:10.1086/381197 14765343

[10] Ho PL , Lam KF , Chow FKH , Lau YL , Wong SSY , Cheng SLE , Chiu SS . 2004. Serotype distribution and antimicrobial resistance patterns of nasopharyngeal and invasive Streptococcus pneumoniae isolates in Hong Kong children. Vaccine 22:3334–3339. doi:10.1016/j.vaccine.2004.02.038 15308357

[11] Dessen A , Mouz N , Gordon E , Hopkins J , Dideberg O . 2001. Crystal structure of Pbp2X from a highly penicillin-resistant Streptococcus pneumoniae clinical isolate: a mosaic framework containing 83 mutations. J Biol Chem 276:45106–45112. doi:10.1074/jbc.M107608200 11553637

[12] Job V , Carapito R , Vernet T , Dessen A , Zapun A . 2008. Common alterations in Pbp1A from resistant Streptococcus pneumoniae decrease its reactivity toward beta-Lactams: structural insights. J Biol Chem 283:4886–4894. doi:10.1074/jbc.M706181200 18055459

[13] Diawara I , Nayme K , Katfy K , Barguigua A , Kettani-Halabi M , Belabbes H , Timinouni M , Zerouali K , Elmdaghri N . 2018. Analysis of amino acid motif of penicillin-binding proteins 1A, 2B, and 2X in invasive Streptococcus pneumoniae nonsusceptible to penicillin isolated from pediatric patients in Casablanca, Morocco. BMC Res Notes 11:632. doi:10.1186/s13104-018-3719-5 30170603 PMC6119257

[14] Li Y , Metcalf BJ , Chochua S , Li Z , Gertz RE , Walker H , Hawkins PA , Tran T , Whitney CG , McGee L , Beall BW . 2016. Penicillin-binding protein transpeptidase signatures for tracking and predicting Β-lactam resistance levels in Streptococcus pneumoniae. mBio 7:e00756-16. doi:10.1128/mBio.00756-16 27302760 PMC4916381

[15] Trzciński K , Thompson CM , Lipsitch M . 2004. Single-step capsular transformation and acquisition of penicillin resistance in Streptococcus pneumoniae. J Bacteriol 186:3447–3452. doi:10.1128/JB.186.11.3447-3452.2004 15150231 PMC415782

[16] Leclercq R . 2002. Mechanisms of resistance to macrolides and Lincosamides: Nature of the resistance elements and their clinical implications. Clin Infect Dis 34:482–492. doi:10.1086/324626 11797175

[17] Epping L , van Tonder AJ , Gladstone RA , Bentley SD , Page AJ , Keane JA . 2018. Seroba: rapid high-throughput serotyping of Streptococcus pneumoniae from whole genome sequence data. Microb Genom 4:e000186. doi:10.1099/mgen.0.000186 29870330 PMC6113868

[18] Gladstone RA , Lo SW , Lees JA , Croucher NJ , van Tonder AJ , Corander J , Page AJ , Marttinen P , Bentley LJ , Ochoa TJ , Ho PL , du Plessis M , Cornick JE , Kwambana-Adams B , Benisty R , Nzenze SA , Madhi SA , Hawkins PA , Everett DB , Antonio M , Dagan R , Klugman KP , von Gottberg A , McGee L , Breiman RF , Bentley SD , Global Pneumococcal Sequencing Consortium . 2019. International genomic definition of Pneumococcal lineages, to contextualise disease, antibiotic resistance and vaccine impact. EBioMedicine 43:338–346. doi:10.1016/j.ebiom.2019.04.021 31003929 PMC6557916

[19] Demczuk W , Martin I , Griffith A , Lefebvre B , McGeer A , Tyrrell GJ , Zhanel GG , Kus JV , Hoang L , Minion J , Van Caeseele P , Gad RR , Haldane D , Zahariadis G , Mead K , Steven L , Strudwick L , Mulvey MR . 2022. Linear regression equations to predict Β-lactam, macrolide, lincosamide, and fluoroquinolone MICs from molecular antimicrobial resistance determinants in Streptococcus pneumoniae. Antimicrob Agents Chemother 66:e0137021. doi:10.1128/AAC.01370-21 34662197 PMC8765234

[20] Snapshot of Australia, 2021 | Australian Bureau of Statistics [Internet]. 2021. Available from: https://www.abs.gov.au/statistics/people/people-and-communities/snapshot-australia/latest-release. Retrieved 5 Apr 2023.

[21] CLSI . 2022. Performance Standards for Antimicrobial Susceptibility Testing, M100. 31. Clinical and Laboratory Standards Institute, Wayne, PA. 10.1128/JCM.00213-21 PMC8601225 34550809

[22] Souvorov A , Agarwala R , Lipman DJ . 2018. SKESA: strategic k-mer extension for scrupulous assemblies. Genome Biol 19:153. doi:10.1186/s13059-018-1540-z 30286803 PMC6172800

[23] Jolley KA , Bray JE , Maiden MCJ . 2018. Open-access bacterial population genomics: BIGSdb software, the PubMLST.org Website and their applications. Wellcome Open Res 3:124. doi:10.12688/wellcomeopenres.14826.1 30345391 PMC6192448

[24] Lees JA , Harris SR , Tonkin-Hill G , Gladstone RA , Lo SW , Weiser JN , Corander J , Bentley SD , Croucher NJ . 2019. Fast and flexible bacterial genomic epidemiology with PopPUNK. Genome Res 29:304–316. doi:10.1101/gr.241455.118 30679308 PMC6360808

[25] Sherry NL , Horan KA , Ballard SA , Gonҫalves da Silva A , Gorrie CL , Schultz MB , Stevens K , Valcanis M , Sait ML , Stinear TP , Howden BP , Seemann T . 2023. An ISO-certified genomics workflow for identification and surveillance of antimicrobial resistance. Nat Commun 14:60. doi:10.1038/s41467-022-35713-4 36599823 PMC9813266

[26] Feldgarden M , Brover V , Haft DH , Prasad AB , Slotta DJ , Tolstoy I , Tyson GH , Zhao S , Hsu C-H , McDermott PF , Tadesse DA , Morales C , Simmons M , Tillman G , Wasilenko J , Folster JP , Klimke W . 2019. Validating the AMRFinder tool and resistance gene database by using antimicrobial resistance genotype-phenotype correlations in a collection of isolates. Antimicrob Agents Chemother 63:e00483-19. doi:10.1128/AAC.00483-19 31427293 PMC6811410

[27] Hunt M , Mather AE , Sánchez-Busó L , Page AJ , Parkhill J , Keane JA , Harris SR . 2017. ARIBA: rapid antimicrobial resistance genotyping directly from sequencing reads. Microb Genom 3:e000131. doi:10.1099/mgen.0.000131 29177089 PMC5695208

[28] Nguyen L-T , Schmidt HA , von Haeseler A , Minh BQ . 2015. IQ-TREE: a fast and effective stochastic algorithm for estimating maximum-likelihood phylogenies. Mol Biol Evol 32:268–274. doi:10.1093/molbev/msu300 25371430 PMC4271533

[29] Fu L , Niu B , Zhu Z , Wu S , Li W . 2012. CD-HIT: accelerated for clustering the next-generation sequencing data. Bioinf 28:3150–3152. doi:10.1093/bioinformatics/bts565 PMC3516142 23060610

[30] Li W , Godzik A . 2006. Cd-hit: a fast program for clustering and comparing large sets of protein or nucleotide sequences. Bioinf 22:1658–1659. doi:10.1093/bioinformatics/btl158 16731699

[31] Edgar RC . 2004. MUSCLE: multiple sequence alignment with high accuracy and high throughput. Nucleic Acids Res 32:1792–1797. doi:10.1093/nar/gkh340 15034147 PMC390337

[32] Le SQ , Gascuel O . 2008. An improved general amino acid replacement matrix. Mol Biol Evol 25:1307–1320. doi:10.1093/molbev/msn067 18367465

[33] Schliep KP . 2011. Phangorn: phylogenetic analysis in R. Bioinf 27:592–593. doi:10.1093/bioinformatics/btq706 PMC3035803 21169378

[34] Yu G , Smith DK , Zhu H , Guan Y , Lam TTY . 2017. Ggtree: an R package for visualization and annotation of phylogenetic trees with their covariates and other associated data. Methods Ecol Evol 8:28–36. doi:10.1111/2041-210X.12628

[35] Zhou L , Feng T , Xu S , Gao F , Lam TT , Wang Q , Wu T , Huang H , Zhan L , Li L , Guan Y , Dai Z , Yu G . 2022. Ggmsa: a visual exploration tool for multiple sequence alignment and associated data. Brief Bioinform 23:bbac222. doi:10.1093/bib/bbac222 35671504

[36] Wickham H , Averick M , Bryan J , Chang W , McGowan L , François R , Grolemund G , Hayes A , Henry L , Hester J , Kuhn M , Pedersen T , Miller E , Bache S , Müller K , Ooms J , Robinson D , Seidel D , Spinu V , Takahashi K , Vaughan D , Wilke C , Woo K , Yutani H . 2019. Welcome to the tidyverse. J Open Source Softw 4:1686. doi:10.21105/joss.01686

[37] CDC . 2019. Antibiotic resistance threats in the United States, 2019. Centre for Dis Control:148.

[38] Tacconelli E , Carrara E , Savoldi A , Harbarth S , Mendelson M , Monnet DL , Pulcini C , Kahlmeter G , Kluytmans J , Carmeli Y , Ouellette M , Outterson K , Patel J , Cavaleri M , Cox EM , Houchens CR , Grayson ML , Hansen P , Singh N , Theuretzbacher U , Magrini N , WHO Pathogens Priority List Working Group . 2018. Discovery, research, and development of new antibiotics: the WHO priority list of antibiotic-resistant bacteria and tuberculosis. Lancet Infect Dis 18:318–327. doi:10.1016/S1473-3099(17)30753-3 29276051

[39] Orio AGA , Piñas GE , Cortes PR , Cian MB , Echenique J , Levin B . 2011. Compensatory evolution of pbp mutations restores the fitness cost imposed by β-lactam resistance in Streptococcus pneumoniae. PLOS Pathog 7:e1002000. doi:10.1371/journal.ppat.1002000 21379570 PMC3040684

[40] Rieux V , Carbon C , Azoulay-Dupuis E . 2001. Complex relationship between acquisition of beta-lactam resistance and loss of virulence in Streptococcus pneumoniae. J Infect Dis 184:66–72. doi:10.1086/320992 11398111

[41] Egorova E , Kumar N , Gladstone RA , Urban Y , Voropaeva E , Chaplin AV , Rumiantseva E , Svistunova TS , Hawkins PA , Klugman KP , Breiman RF , McGee L , Bentley SD , Lo SW . 2022. Key features of pneumococcal isolates recovered in central and Northwestern Russia in 2011–2018 determined through whole-genome sequencing. Microb Genom 8:mgen000851. doi:10.1099/mgen.0.000851 36112007 PMC9676041

[42] Rockett RJ , Oftadeh S , Bachmann NL , Timms VJ , Kong F , Gilbert GL , Sintchenko V . 2018. Genome-wide analysis of Streptococcus pneumoniae serogroup 19 in the decade after the introduction of pneumococcal conjugate vaccines in Australia. Sci Rep 8:16969. doi:10.1038/s41598-018-35270-1 30446692 PMC6240094

[43] van Tonder AJ , Bray JE , Quirk SJ , Haraldsson G , Jolley KA , Maiden MCJ , Hoffmann S , Bentley SD , Haraldsson Á , Erlendsdóttir H , Kristinsson KG , Brueggemann AB . 2016. Putatively novel serotypes and the potential for reduced vaccine effectiveness: capsular locus diversity revealed among 5405 pneumococcal genomes. Microb Genom 2:000090. doi:10.1099/mgen.0.000090 28133541 PMC5266551

[44] Tran T-H , Kwon H-Y , Kim E-H , Kim K-W , Briles DE , Pyo S , Rhee D-K . 2011. Decrease in penicillin susceptibility due to heat shock protein ClpL in Streptococcus pneumoniae. Antimicrob Agents Chemother 55:2714–2728. doi:10.1128/AAC.01383-10 21422206 PMC3101445

