# Supplementary material for: Comparison of contemporary invasive and non-invasive Streptococcus pneumoniae isolates reveals new insights into circulating anti-microbial resistance determinants
Source: Antimicrob Agents Chemother. 2023 Oct 12;67(11):e00785-23. doi: 10.1128/aac.00785-23 (PMC10649040; doi:10.1128/aac.00785-23)
Supplement: Fig S1 — All supplemental figures. [file aac.00785-23-s0001.pdf]

Supplementary Materials for

Comparison of contemporary invasive and non-invasive *Streptococcus pneumoniae* isolates reveals new insights into circulating antimicrobial resistance determinants

Charlie Higgs, Lamali Sadeesh Kumar, Kerrie Stevens, Janet Strachan, Tony Korman, Kristy Horan, Diane Daniel, Madeline Russell, Christopher A. McDevitt, Norelle L. Sherry, Timothy P. Stinear, Benjamin P. Howden, Claire L. Gorrie

The Supplementary Materials include:

Supplementary Table 1 – 2
Supplementary Figures 1 – 9
Legends for Supplementary Data 1

Other Supplementary Materials for this manuscript include the following:

Supplementary Data 1

Supplementary tables and figures:

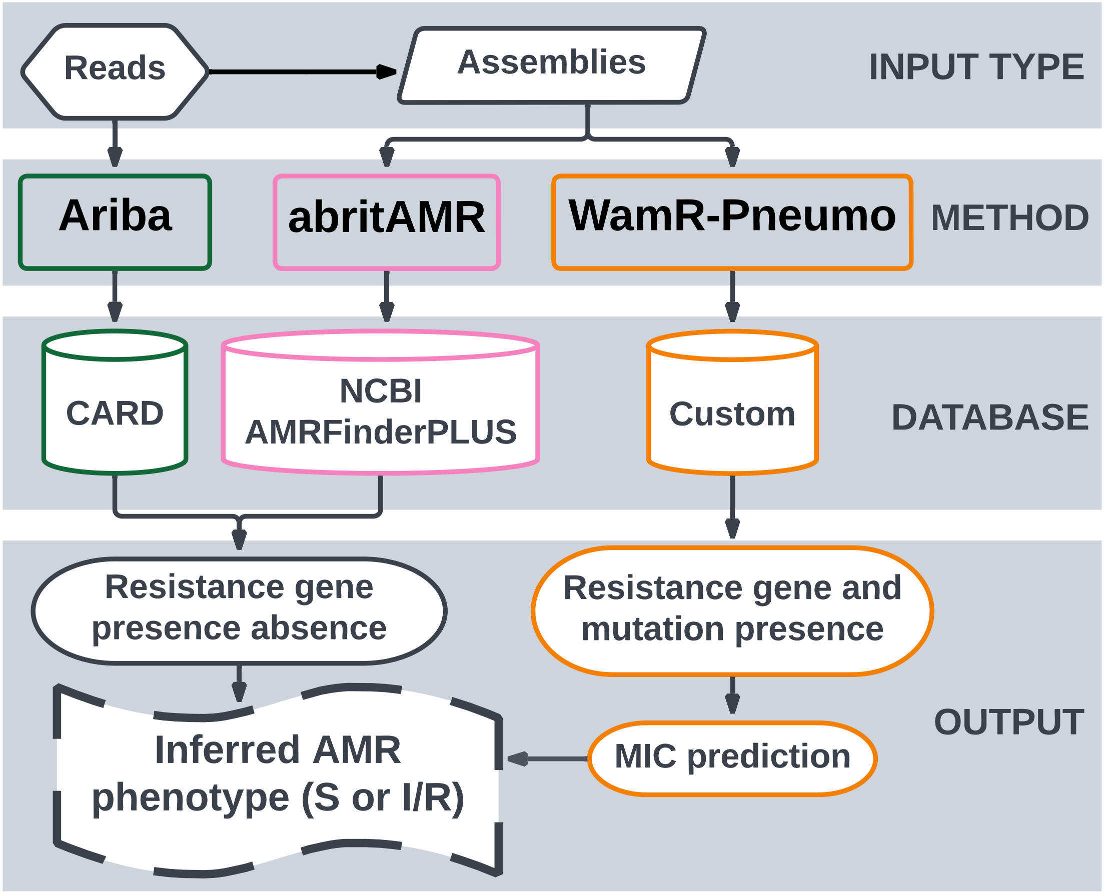

Supplementary Figure 1: Workflow detailing the *in silico* AMR prediction tools used and the data bases that each tool uses.

**Population**	**Number of isolates resistant to at least one antimicrobial class** (% total)	**p value** (proportion test)	**Number of isolates multidrug resistant** (% total)	**p value** (proportion test)	**Proportion of isolates resistant to one antimicrobial class that are also multidrug resistant**	**p value** (proportion test)
**Invasive** (n=1288)	308 (23.9)	*NS*	59 (4.6)	p<0.05	19.2%	p<0.01
**Non-invasive** (n=186)	54 (29.0)		19 (10.2)		35.2%	

Supplementary Table 1: Comparison of the proportion of isolates that are multidrug resistant.

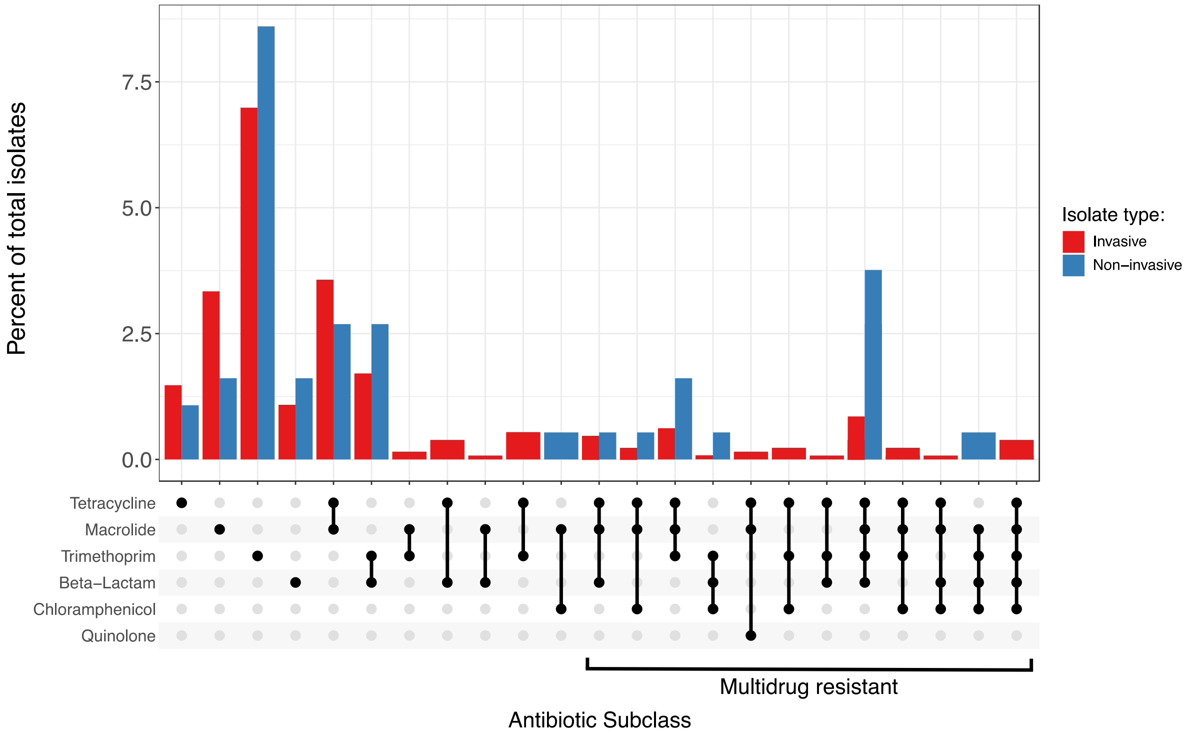

Supplementary Figure 2: Cooccurrence of resistance to each antimicrobial class. Percent of total isolates is calculated based on the number of isolates in each population; invasive n=1,288 and non-invasive n=186. Multidrug resistance was defined as isolates that are resistant to 3 or more classes of antibiotics. If multiple breakpoints existed for an antibiotic, the most stringent were used (i.e. meningitis or oral).

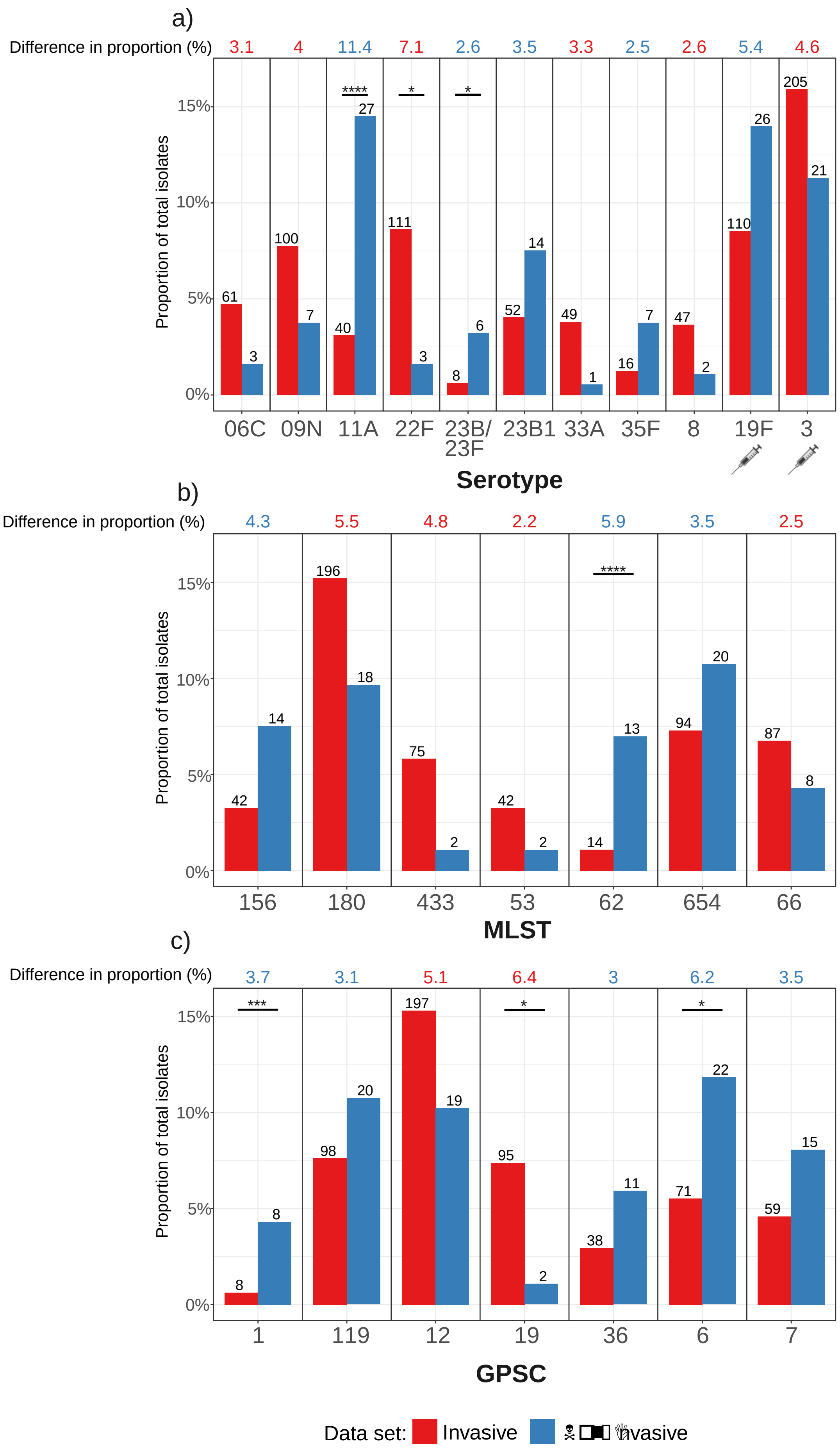

a)
Difference in proportion (%)
3.1
4
11.4
7.1
2.6
3.5
3.3
2.5
2.6
5.4
4.6
Proportion of total isolates
0%
5%
10%
15%
****
*
*
61
3
100
7
40
27
111
3
8
6
52
14
49
1
16
7
47
2
110
26
205
21
06C
09N
11A
22F
23B/
23F
23B1
33A
35F
8
19F
3
Serotype
b)
Difference in proportion (%)
4.3
5.5
4.8
2.2
5.9
3.5
2.5
****
42
14
196
18
75
2
42
2
14
13
94
20
87
8
156
180
433
53
62
654
66
MLST
c)
Difference in proportion (%)
3.7
3.1
5.1
6.4
3
6.2
3.5
***
*
*
8
8
98
20
197
19
95
2
38
11
71
22
59
15
1
119
12
19
36
6
7
GPSC
Data set:
Invasive

Supplementary Figure 3: Types/clusters overrepresented in each data set. Isolates typed based on a) serotype, b) multi-locus sequence typing (MLST) and c) global pneumococcal sequence cluster (GPSC). Only types/clusters that had a difference of >2% in the relative proportions in each data set are displayed. The difference in proportion between the two data sets is shown above the bar charts and is coloured based on the data set that the type/cluster is more prevalent in. Serotypes contained in the 13-valent pneumococcal conjugate vaccine are indicated using a vaccine icon. A count of isolates for each typing/clustering method is displayed at the top of each bar in black text. p values have been indicated only if they were ≤ a 0.05 significance threshold. Total number of isolates in each data set: invasive (n=1,288) and non-invasive (n=186).

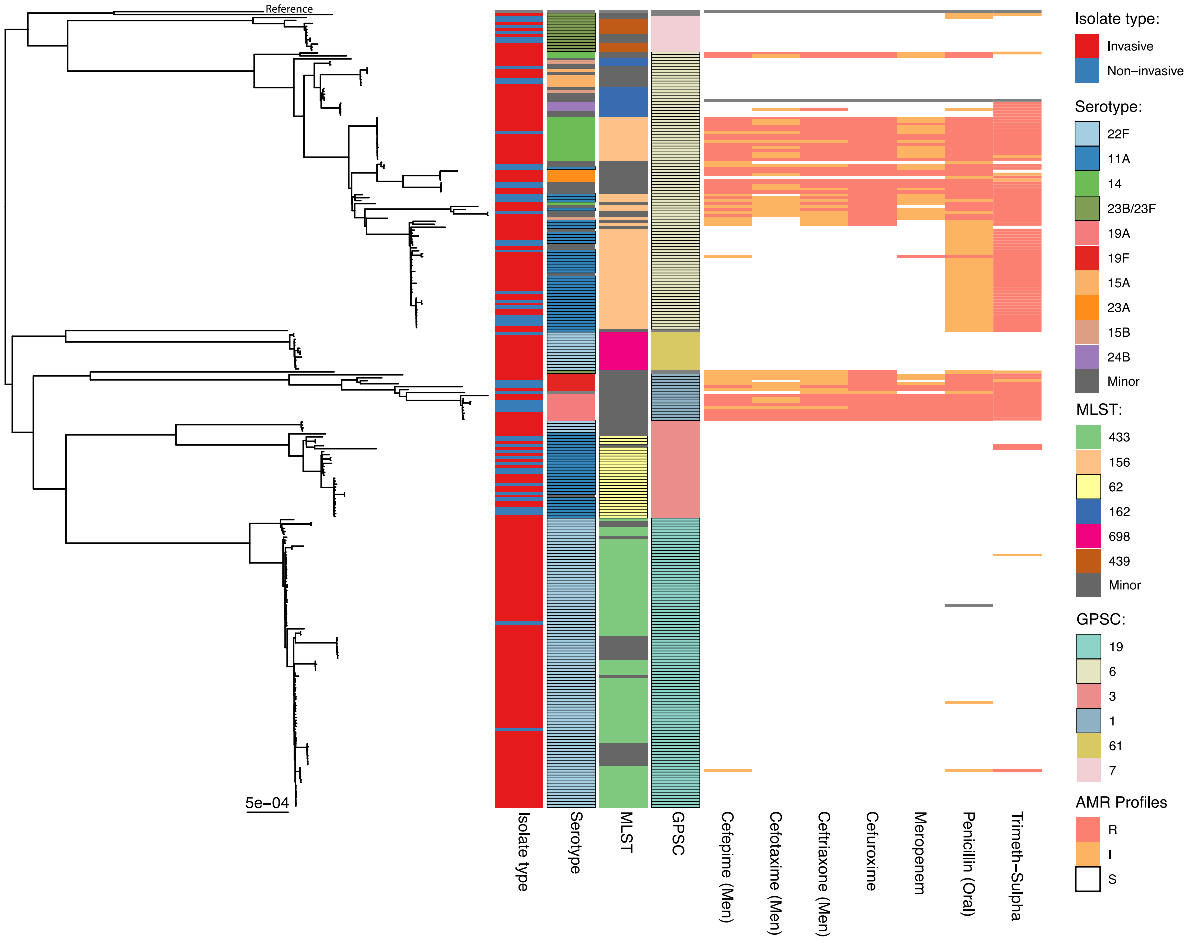

Supplementary Figure 4: Midpoint rooted phylogenetic tree of all significantly overrepresented types/clusters (n=269). Included in the tree are all serotypes 11A, 22F and 23B/23F, ST 62 and GPSCs 1, 19 and 6 isolates. These types/clusters have also been outlined in black in the heatmap to show they are significantly different between the two populations. Minor serotypes, multi-locus sequence types (MLSTs) and global pneumococcal sequence cluster (GPSC) were defined as those that contained less than 10 isolates over the study period. The reference was ASM966447v1 (GCA_009664475.1); collected 2014, serotype 19A, ST 199.

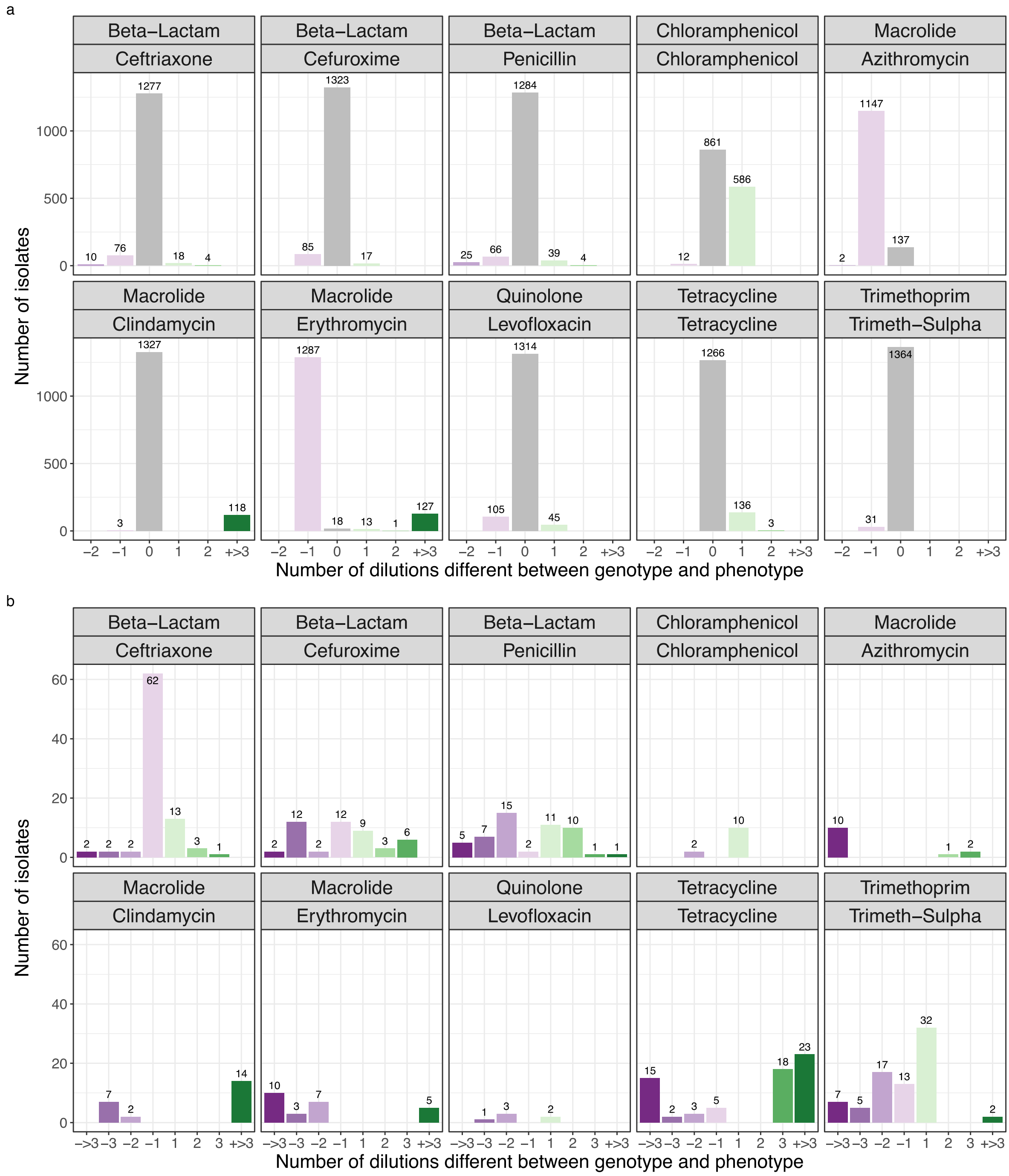

Supplementary Figure 5: Number of dilutions different between antimicrobial MIC phenotype and genotype (WamR-Pneumo). MICs have been separated into a) when the phenotype and genotype had the same MIC interpretation and b) had different MIC interpretations. Interpretations are from the 2022 CLSI guidelines using oral breakpoints for penicillin and meningitis breakpoints for ceftriaxone.

Antibiotic to which gene contribute to resistance	Gene	Number of unique alleles identified in both populations (DNA)	Number of unique alleles identified in both populations (AA)	Number of alleles found in ≥1 isolate with ≥1 dilutions different between phenotypic and genotypic MIC
Beta-Lactam	pbp1a	127	78	42
Beta-Lactam	pbp2b	155	102	57
Beta-Lactam	pbp2x	173	126	75

Supplementary Table 2: Number of alleles of key resistance genes identified in the study data set.

a)

Penicillin MIC (mg/L) = 2^[round (– 4.61 +
(1.547 × PBP1a (motif 1) – STMK → any) +
(0.949 × PBP1a (motif 4) – TSQF → any) +
(1.202 × PBP2b (motif 2) – SSNT → any) +
(0.356 × PBP2b (motif 3) – QLQPT → any) +
(1.626 × PBP2x (motif 1) – STMK → SAFK) +
(1.548 × PBP2x (motif 3) – KDA → EDT) +
(0.680 × PBP2x (motif 3) – KDA → KEA) +
(0.753 × PBP2x (motif 4) – LKSG → VKSG)]

b)

Cefuroxime MIC (mg/L) = 2^[round (– 1.018 +
(1.509 × PBP1a (motif 1) – STMK → SAMK) +
(2.170 × PBP1a (motif 1) – STMK → SSMK) +
(2.322 × PBP2x (motif 1) – STMK → SAFK) +
(0.256 × PBP2x (motif 1) – STMK → other) +
(1.026 × PBP2x (motif 4) – LKSG → VKSG)]

c)

Ceftriaxone MIC (mg/L) = 2^[round (– 2.709 +
(1.25 × PBP1a (motif 1) – STMK → any) +
(2.72 × PBP2x (motif 1) – STMK → SAFK) +
(0.76 × PBP2x (motif 3) – KDA → EDT) +
(0.989 × PBP2x (motif 4) – LKSG → VKSG)]

Supplementary Figure 6: Regression model equations used by WamR-Pneumo to predict isolates beta-lactam MIC values. a) Penicillin, b) Cefuroxime and c) Ceftriaxone. These equations were developed by Demczuk *et al.*, Antimicrobial Agents and Chemotherapy, 2022 (https://pubmed.ncbi.nlm.nih.gov/34662197/)

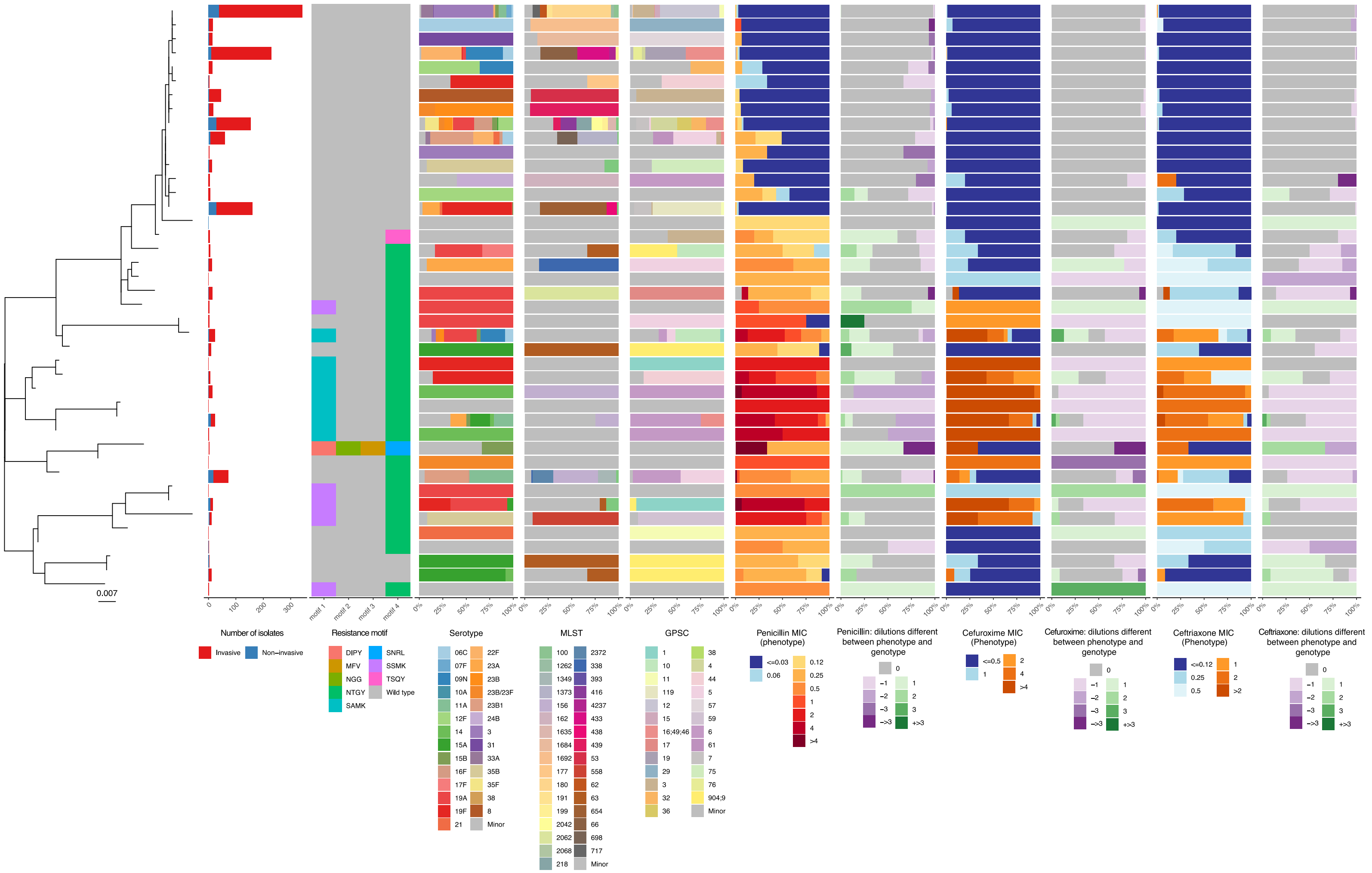
0.007
Number of isolates
0
100
200
300
Invasive
Non−invasive
Resistance motif
motif 1
motif 2
motif 3
motif 4
DIPY
MFV
NGG
NTGY
SAMK
SNRL
SSMK
TSQY
Wild type
Serotype
06C
07F
09N
10A
11A
12F
14
15A
15B
16F
17F
19A
19F
21
22F
23A
23B
23B/23F
23B1
24B
3
31
33A
35B
35F
38
8
Minor
MLST
100
1262
1349
1373
156
162
1635
1684
1692
177
180
191
199
2042
2062
2068
218
2372
338
393
416
4237
433
438
439
53
558
62
63
654
66
698
717
Minor
GPSC
1
10
11
119
12
15
16;49;46
17
19
29
3
32
36
38
4
44
5
57
59
6
61
7
75
76
904;9
Minor
Penicillin MIC (phenotype)
<=0.03
0.06
0.12
0.25
0.5
1
2
4
>4
Penicillin: dilutions different between phenotype and genotype
0
−1
−2
−3
−>3
1
2
3
+>3
Cefuroxime MIC (Phenotype)
<=0.5
1
2
4
>4
Cefuroxime: dilutions different between phenotype and genotype
0
−1
−2
−3
−>3
1
2
3
+>3
Ceftriaxone MIC (Phenotype)
<=0.12
0.25
0.5
1
2
>2
Ceftriaxone: dilutions different between phenotype and genotype
0
−1
−2
−3
−>3
1
2
3
+>3
0%
25%
50%
75%
100%

Supplementary Figure 7: Midpoint rooted maximum likelihood phylogenetic tree of PBP1a. Tree has been built using an amino acid sequence alignment of all unique proteins that had at least one isolate with at least one dilution difference between the phenotypic and genotypic MIC. The number of isolates is based on counts of isolates that had contained the PBP1a alleles. The resistance motifs are the motifs used by WamR-Pneumo to determine the beta-lactam MIC. The MIC panels (Penicillin (oral breakpoints), cefuroxime (meningitis breakpoints) and ceftriaxone (meningitis breakpoints)) show the proportion of isolates with each MIC for each of the PBP2x alleles (blue for susceptible and orange/red for intermediate/resistant. The panels displaying the number of dilutions different between the phenotype and genotype uses WamR-Pneumo to predict the MIC using genotype.

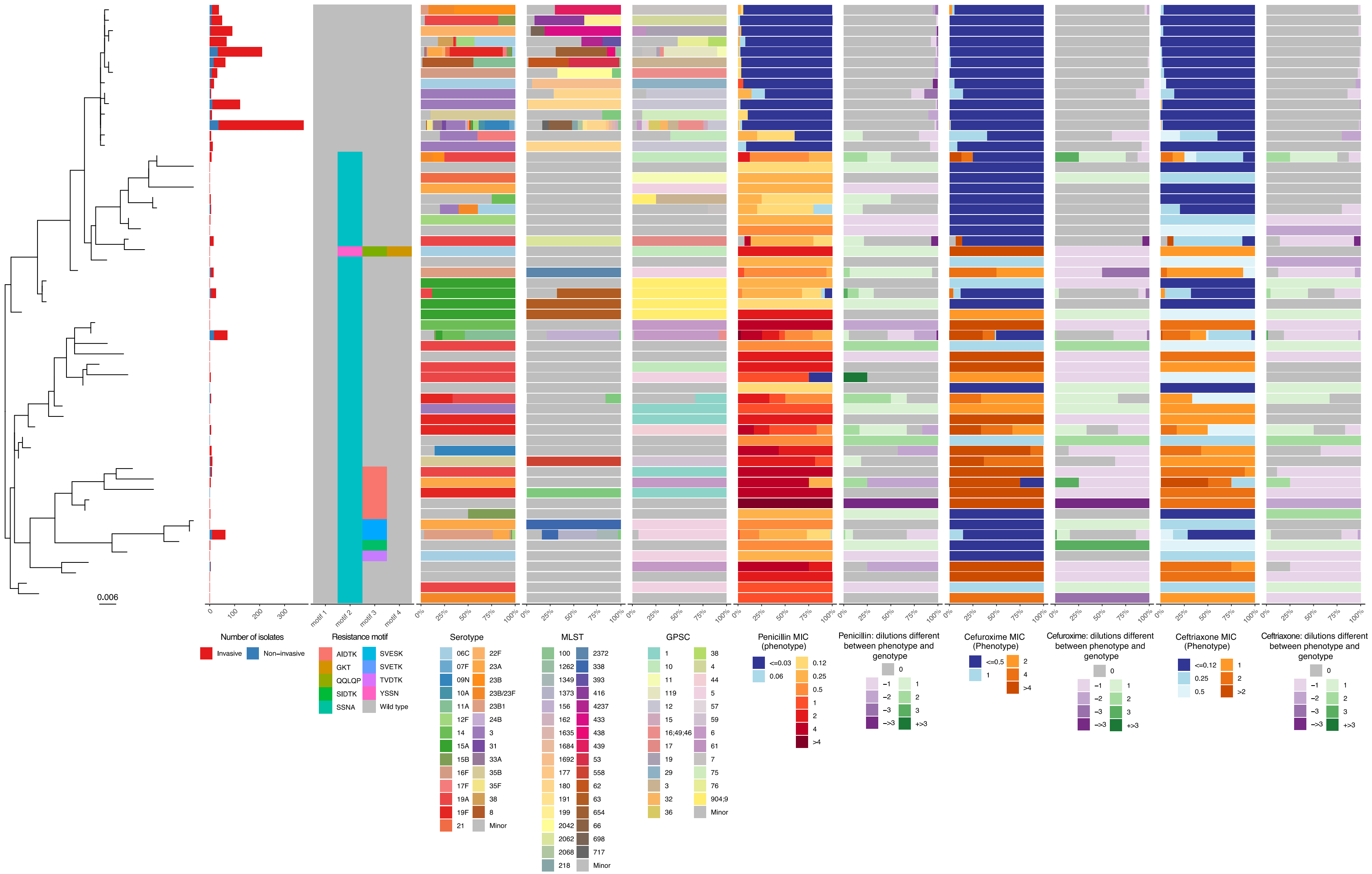

Supplementary Figure 8: Midpoint rooted maximum likelihood phylogenetic tree of PBP2b. Tree has been built using an amino acid sequence alignment of all unique proteins that had at least one isolate with at least one dilution difference between the phenotypic and genotypic MIC. The number of isolates is based on counts of isolates that had contained the PBP2b alleles. The

resistance motifs are the motifs used by WamR-Pneumo to determine the beta-lactam MIC. The MIC panels (Penicillin (oral breakpoints), cefuroxime (meningitis breakpoints) and ceftriaxone (meningitis breakpoints)) show the proportion of isolates with each MIC for each of the PBP2x alleles (blue for susceptible and orange/red for intermediate/resistant. The panels displaying the number of dilutions different between the phenotype and genotype uses WamR-Pneumo to predict the MIC using genotype.

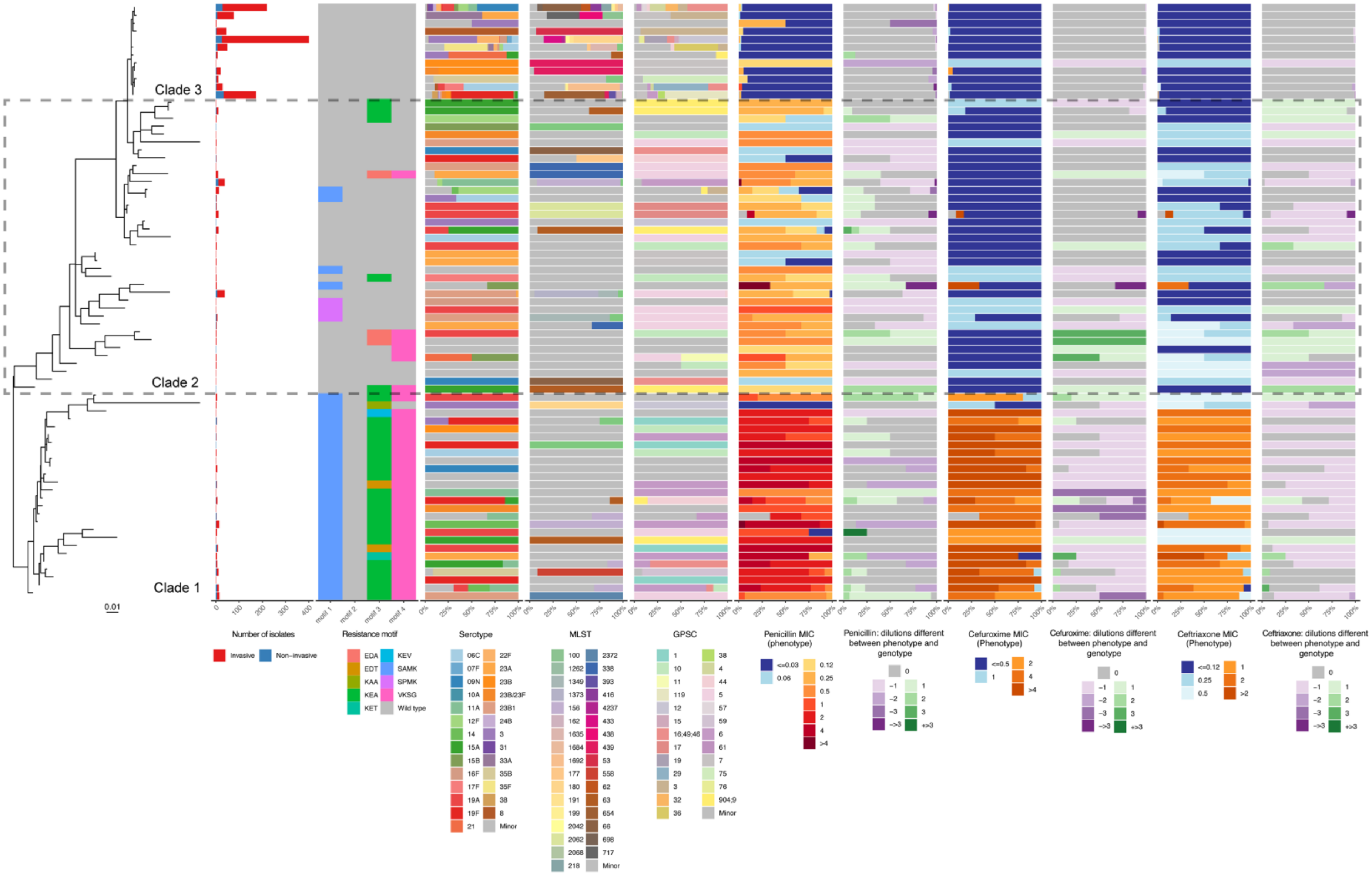
Clade 3
Clade 2
Clade 1
0.01
Number of isolates
Invasive
Non−invasive
Resistance motif
motif 1
motif 2
motif 3
motif 4
EDA
EDT
KAA
KEA
KET
KEV
SAMK
SPMK
VKSG
Wild type
Serotype
06C 07F 09N 10A 11A 12F 14 15A 15B 16F 17F 19A 19F 21
22F 23A 23B 23B/23F 23B1 24B 3 31 33A 35B 35F 38 8 Minor
MLST
100 1262 1349 1373 156 162 1635 1684 1692 177 180 191 199 2042 2062 2068 218
2372 338 393 416 4237 433 438 439 53 558 62 63 654 66 698 717 Minor
GPSC
1 10 11 119 12 15 16;49;46 17 19 29 3 32 36
38 4 44 5 57 59 6 61 7 75 76 904;9 Minor
Penicillin MIC (phenotype)
<=0.03 0.06 0.12 0.25 0.5 1 2 4 >4
Penicillin: dilutions different between phenotype and genotype
0 −1 −2 −3 −>3 1 2 3 +>3
Cefuroxime MIC (Phenotype)
<=0.5 1 2 4 >4
Cefuroxime: dilutions different between phenotype and genotype
0 −1 −2 −3 −>3 1 2 3 +>3
Ceftriaxone MIC (Phenotype)
<=0.12 0.25 0.5 1 2 >2
Ceftriaxone: dilutions different between phenotype and genotype
0 −1 −2 −3 −>3 1 2 3 +>3

Supplementary Figure 9: Midpoint rooted maximum likelihood phylogenetic tree of PBP2x. Tree has been built using an amino acid sequence alignment of all unique proteins that had at least one isolate with at least one dilution difference between the phenotypic and genotypic MIC. The number of isolates is based on counts of isolates that had contained the PBP2x alleles. The resistance motifs are the motifs used by WamR-Pneumo to determine the beta-lactam MIC. The MIC panels (Penicillin (oral breakpoints), cefuroxime (meningitis breakpoints) and ceftriaxone (meningitis breakpoints)) show the proportion of isolates with each MIC for each of the PBP2x alleles (blue for susceptible and orange/red for intermediate/resistant. The panels displaying the number of dilutions different between the phenotype and genotype uses WamR-Pneumo to predict the MIC using genotype.

Supplementary data:

Supplementary Data 1: Isolate list and metadata for all isolates, including AST results.

Supplementary Data 2: Results from in silico AMR prediction comparison.
